# Atypical measures of diffusion at the gray‐white matter boundary in autism spectrum disorder in adulthood

**DOI:** 10.1002/hbm.25237

**Published:** 2020-10-23

**Authors:** Anke Bletsch, Tim Schäfer, Caroline Mann, Derek S. Andrews, Eileen Daly, Maria Gudbrandsen, Amber N. V. Ruigrok, Robert Dallyn, Rafael Romero‐Garcia, Meng‐Chuan Lai, Michael V. Lombardo, Michael C. Craig, John Suckling, Edward T. Bullmore, Simon Baron‐Cohen, the MRC AIMS Consortium, Declan G. M. Murphy, Flavio Dell'Acqua, Christine Ecker

**Affiliations:** ^1^ Department of Child and Adolescent Psychiatry, Psychosomatics and Psychotherapy University Hospital, Goethe University Frankfurt Germany; ^2^ Department of Psychiatry and Behavioral Sciences at the M.I.N.D. Institute University of California Davis California USA; ^3^ Department of Forensic and Neurodevelopmental Sciences, and the Sackler Institute for Translational Neurodevelopmental Sciences Institute of Psychiatry, Psychology and Neuroscience, King's College London UK; ^4^ Autism Research Centre, Department of Psychiatry University of Cambridge Cambridge UK; ^5^ Brain Mapping Unit, Department of Psychiatry University of Cambridge Cambridge UK; ^6^ Centre for Addiction and Mental Health and The Hospital for Sick Children, Department of Psychiatry University of Toronto Toronto Canada; ^7^ Department of Psychiatry National Taiwan University Hospital and College of Medicine Taipei Taiwan; ^8^ Laboratory for Autism and Neurodevelopmental Disorders Center for Neuroscience and Cognitive Systems, Istituto Italiano di Tecnologia Rovereto Italy; ^9^ National Autism Unit Bethlem Royal Hospital London UK

**Keywords:** diffusion tensor imaging, multimodal imaging, myelinated and unmyelinated gray matter, superficial white matter

## Abstract

Autism spectrum disorder (ASD) is a highly complex neurodevelopmental condition that is accompanied by neuroanatomical differences on the macroscopic and microscopic level. Findings from histological, genetic, and more recently in vivo neuroimaging studies converge in suggesting that neuroanatomical abnormalities, specifically around the gray‐white matter (GWM) boundary, represent a crucial feature of ASD. However, no research has yet characterized the GWM boundary in ASD based on measures of diffusion. Here, we registered diffusion tensor imaging data to the structural T1‐weighted images of 92 adults with ASD and 92 matched neurotypical controls in order to examine between‐group differences and group‐by‐sex interactions in fractional anisotropy and mean diffusivity sampled at the GWM boundary, and at different sampling depths within the superficial white and into the gray matter. As hypothesized, we observed atypical diffusion at and around the GWM boundary in ASD, with between‐group differences and group‐by‐sex interactions depending on tissue class and sampling depth. Furthermore, we identified that altered diffusion at the GWM boundary partially (i.e., ~50%) overlapped with atypical gray‐white matter tissue contrast in ASD. Our study thus replicates and extends previous work highlighting the GWM boundary as a crucial target of neuropathology in ASD, and guides future work elucidating etiological mechanisms.

## Graphical abstract

The present study observed atypical diffusion at and around the gray‐white matter (GWM) boundary in individuals with autism spectrum disorder (ASD) relative to neurotypical controls, with between‐group differences and group‐by‐sex interactions depending on tissue class and sampling depth. Altered diffusion at the GWM boundary was further identified to partially (i.e., ~50%) and nonrandomly overlap with atypical gray‐white matter tissue contrast in ASD. The study thus replicates and extends previous work highlighting the GWM boundary as a crucial target of neuropathology in ASD, and guides future work elucidating etiological mechanisms.

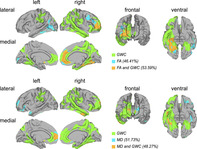

## 1 INTRODUCTION

Autism spectrum disorder (ASD) is a highly complex neurodevelopmental condition characterized by impaired social reciprocity and communication, as well as repetitive behaviors (Wing, 1997). Evidence suggests that ASD is accompanied by differences in brain anatomy and connectivity, on the macroscopic and microscopic level, that include atypicalities at the gray‐white matter (GWM) boundary. For example, results from histological (Avino & Hutsler, 2010) and in vivo neuroimaging studies (Andrews et al., 2017; Bezgin, Lewis, & Evans, 2018; Hong, Valk, Di Martino, Milham, & Bernhardt, 2018; Mann et al., 2018) suggest that the GWM boundary is less well defined in ASD. This indistinct boundary is potentially due to an abnormal cell patterning at the GWM transition zone (Avino & Hutsler, 2010), with an excess of interstitial neurons distributed among the cortico‐cortical U‐fibers of the superficial white matter (Avino & Hutsler, 2010; Bailey et al., 1998; Casanova, Buxhoeveden, Switala, & Roy, 2002; Hutsler, Love, & Zhang, 2007; Simms, Kemper, Timbie, Bauman, & Blatt, 2009; Wegiel et al., 2010), which may result from deviations in fetal neuronal proliferation or decreased developmental apoptosis (Avino & Hutsler, 2010; Chun & Shatz, 1989; for a review, see McFadden & Minshew, 2013). Moreover, abnormalities at and around the GWM boundary have been inferred from a reduced gray‐white matter tissue contrast (GWC) in ASD, which may be driven by increased T1‐weighted signal intensity within the gray matter (Andrews et al., 2017). However, it is currently unknown whether this increase in signal intensity arises from alterations in myelination and/or differences in the underlying gray matter cytoarchitecture. Here, we examined the GWM boundary and adjacent white and gray matter in ASD based on measures of diffusion.

Diffusion metrics that utilize tensor‐based models (DTI), such as fractional anisotropy (FA) and mean diffusivity (MD), are particularly sensitive to white matter neuroarchitecture and changes thereof (Beaulieu, 2002; Jbabdi, Sotiropoulos, Haber, Van Essen, & Behrens, 2015; Jones, Knösche, & Turner, 2013). To date, DTI studies in ASD have predominantly focused on examining large‐scale brain anatomical connectivity based on fiber tracts within the deep white matter (for reviews, see Aoki, Abe, Nippashi, & Yamasue, 2013; Catani et al., 2016; Travers et al., 2012). Few studies have examined the superficial white matter in ASD, which is located directly beneath the GWM boundary and predominantly consists of short‐range association fibers. Nevertheless, those which are available observed decreased FA in the frontal lobe and increased MD in frontal, temporal, and parietal lobes (Shukla, Keehn, Smylie, & Müller, 2011; Sundaram et al., 2008). Consistent with these results, a recent study highlighted the potentially important role of aberrant white matter neuroarchitecture in the superficial white matter in ASD by linking it to atypical functional connectivity and symptom severity (Hong, Hyung, Paquola, & Bernhardt, 2019). Deviations within the superficial white matter have also been reported by postmortem studies; these reported differences in both the density and thickness of myelinated axons underneath the anterior cingulate and the lateral prefrontal cortices in ASD (Zikopoulos, Liu, et al., 2018; Zikopoulos & Barbas, 2010; Zikopoulos, García‐Cabezas, & Barbas, 2018). Thus, there is increasing evidence to support the suggestion that measures of diffusion within the superficial white matter are significantly altered in ASD (in addition to abnormalities within the deeper white matter). However, it is unknown if atypical diffusion within the superficial white matter in ASD corresponds to aberrant diffusion at the adjacent GWM boundary, and/or within the cortical gray matter.

The interpretation of diffusion metrics sampled within the cortical gray matter is challenging due to its highly complex composition in terms of both myelo‐ and cytoarchitecture (for a review, see Roebroeck, Miller, & Aggarwal, 2019). For example, based on its myelin content, cortical gray matter can be further subdivided into myelinated and unmyelinated parts, with axons in the deeper layers being predominantly myelinated, and axons in the outer (i.e., more superficial) cortical layers being only lightly—or not at all—myelinated (Geyer, Weiss, Reimann, Lohmann, & Turner, 2011; Nave & Werner, 2014; Rowley et al., 2015; Sprooten et al., 2019). Intracortical myelin maturation has further been shown to extend into adulthood (Grydeland, Walhovd, Tamnes, Westlye, & Fjell, 2013; Rowley et al., 2017; Shafee, Buckner, & Fischl, 2015) and the strongest developmental effect of age‐related increases was observed around the internal layer of projection neurons (Whitaker et al., 2016). A number of recent neuroimaging studies have therefore applied DTI to examine the laminar structure of the cortical gray matter in (postmortem tissue of) healthy humans ex vivo (Aggarwal, Nauen, Troncoso, & Mori, 2015; Bastiani et al., 2016; Dell'Acqua, Bodi, Slater, Catani, & Modo, 2013; Kleinnijenhuis et al., 2013; Leuze et al., 2014) and in vivo (Kleinnijenhuis et al., 2015; McNab et al., 2013). In ASD, however, studies utilizing measures of diffusion to examine the cortical gray matter are scarce and only included small samples of children and adolescents with ASD as compared to typically developing (TD) controls. These studies reported widespread reductions in FA of the gray matter in young children with ASD (Pichiecchio et al., 2016), decreases in MD in frontal and insular regions and increases in MD in occipital regions of the gray matter in older children with ASD (Li, Xue, Ellmore, Frye, & Wong, 2013), and widespread MD increases of the gray matter in adolescents with ASD (Groen, Buitelaar, van der Gaag, & Zwiers, 2011). In adult individuals with ASD, however, to our best knowledge, cortical gray matter has not yet been assessed based on measures of diffusion.

Thus, the objectives of our study were to (a) characterize the GWM boundary based on measures of diffusion in individuals with ASD relative to TD controls, and (b) identify the tissue and depthdependency of diffusion alterations in ASD within the adjacent superficial white and gray matter. Moreover, we aimed to (c) establish whether diffusion alterations in ASD are modulated by sex (i.e., group‐by‐sex interactions), and (d) identify how variability in measures of diffusion at the GWM boundary relates to interindividual differences in GWC in ASD. Based on previous research, we expected individuals with ASD to have significant differences in measures of diffusion at and around the GWM boundary compared to TD controls. Furthermore, we predicted at least a partial spatial overlap of between‐group differences in diffusion properties and in GWC at the GWM transition zone.

## 2 MATERIALS AND METHODS

### 2.1 Participants

Overall, 92 right‐handed adults with ASD (53 males and 39 females) and 92 age, sex, and IQ matched right‐handed TD controls (51 males and 41 females) aged 18–52 years were assessed at the Institute of Psychiatry, Psychology, and Neuroscience (IoPPN), London, and the Autism Research Centre, Cambridge. Approximately equal ratios of ASD to TD individuals, as well as males to females, were recruited within sites (see Table 1). Females and males were recruited in approximately equal numbers across groups, which also allowed us to examine sex‐differences in the brain in ASD. ASD diagnosis was made by a consultant psychiatrist using ICD‐10 research diagnostic criteria and confirmed using the Autism Diagnostic Interview‐Revised (ADI‐R; Lord, Rutter, & Le Couteur, 1994). The ADI‐R rather than the Autism Diagnostic Observation Schedule (ADOS; Lord et al., 2000) was employed as inclusion criteria to ensure that all participants with ASD met the criteria for childhood autism. ADI‐R interviews were completed for 88 individuals with ASD (53 males and 35 females), out of which 73 individuals (47 males and 26 females) reached algorithm cut‐offs for autism in all three domains (social, communication, and restricted/stereotyped behavior). Although failure to reach cut‐off in one domain was permitted (i.e., by one point in the social or communication domain, or alternatively by two points in the repetitive behavior domain) and applied to the remaining 15 participants (6 males and 9 females). We were unable to complete ADI‐R for four females with ASD as their parents/caregivers were not available. However, all of them reached algorithm cutoffs for “autism spectrum” on the ADOS module 4 (social and communication total score) diagnostic algorithm. In all other participants, ADOS scores were used to measure current symptoms and not as an inclusion criterion.

**TABLE 1:** Participant demographics and global brain measures

	ASD (*n* = 92)	TD controls (*n* = 92)	Test statistic	
			*χ* ^2^/*t*	*p*
Age (years)	26.71 ± 7.20 (18–48)	28.38 ± 6.73 (18–52)	−1.62	.11
Sex (male/female)	53/39	51/41	0.02	.88
Site (London/Cambridge)	42/50	47/45	0.35	.56
Sex by site				
Male (London/Cambridge)	29/24	30/21	0.05	.82
Female (London/Cambridge)	13/26	17/24	0.27	.60
WASI (FSIQ)	113.48 ± 12.89 (83–136)	116.40 ± 10.39 (93–137)	−1.69	.09
ADI‐R *Social Interaction*	17 ± 5 (9–28)	—	—	—
ADI‐R *Communication*	13 ± 4 (7–24)	—	—	—
ADI‐R *Repetitive Behavior*	5 ± 2 (1–10)	—	—	—
ADOS	8 ± 5 (0–18)	—	—	—
Total brain volume (l)	1.25 ± 0.14 (1.01–1.72)	1.24 ± 0.12 (0.95–1.48)	0.81	.42
Total cortical volume (l)	0.78 ± 0.08 (0.62–1.06)	0.76 ± 0.07 (0.61–0.90)	1.21	.23
Total gray matter volume (l)	0.60 ± 0.07 (0.48–0.83)	0.60 ± 0.05 (0.47–0.71)	1.29	.20
Total white matter volume (l)	0.47 ± 0.06 (0.35–0.66)	0.47 ± 0.06 (0.34–0.59)	0.23	.82
Total surface area (m^2^)	0.19 ± 0.02 (0.15–0.26)	0.19 ± 0.02 (0.15–0.23)	0.87	.38
Mean cortical thickness (mm)	2.67 ± 0.09 (2.46–2.89)	2.66 ± 0.08 (2.45–2.88)	0.51	.61

*Note:* Data expressed as mean ± *SD* (range).

Abbreviations: ADI‐R, Autism Diagnostic Interview‐Revised; ADOS, Autism Diagnostic Observation Schedule; ASD: autism spectrum disorder; TD: typically developing; FSIQ: full‐scale intelligence quotient; WASI, Wechsler Abbreviated Scale of Intelligence.

Exclusion criteria included a history of major psychiatric disorder (e.g., psychosis), head injury, genetic disorder associated with autism (e.g., fragile‐X syndrome, tuberous sclerosis), or any other medical condition affecting brain function (e.g., epilepsy). Participants taking antipsychotic medication, mood stabilizers, or benzodiazepines were also excluded. A small number of included participants with ASD took antidepressant medication (*n* = 7), out of which *n* = 5 participants also had a comorbid clinical diagnosis of depression. In addition, *n* = 4 other ASD participants had a comorbid diagnosis of depression but did not take any medication. In all participants, the Beck Depression Inventory (BDI; Beck & Steer, 1987) was administered to assess severity of depressive symptoms. In addition, to provide a comprehensive clinical characterization of the assessed sample, several clinical questionnaires were administered in all participants focusing on core autistic symptoms and traits (i.e., Autism Spectrum Quotient [AQ]; Baron‐Cohen, Wheelwright, Skinner, Martin, & Clubley, 2001), including empathy (i.e., Empathy Quotient; Baron‐Cohen & Wheelwright, 2004) and systemizing abilities (i.e., Systemizing Quotient; Baron‐Cohen, Richler, Bisarya, Gurunathan, & Wheelwright, 2003), as well as assessing common comorbidities, that is, anxiety (i.e., Beck Anxiety Inventory [BAI]; Beck & Steer, 1993) and obsessive and compulsive behaviors (i.e., Obsessive–Compulsive Inventory‐Revised; Abramowitz & Deacon, 2006; Huppert et al., 2007). Information on these clinical characteristics of the sample is provided in the Supplementary Material (see Supplementary Table S1). Overall intellectual ability was assessed using the Wechsler Abbreviated Scale of Intelligence (Wechsler, 1999) and all included participants have a full‐scale IQ (FSIQ) >70 (see Table 1). Informed written consent was given by all participants in accordance with ethics approval by the National Research Ethics Committee, Suffolk, UK (Ref: 04/Q0102/26).

### 2.2 MRI data acquisition

All participants were scanned with contemporary magnetic resonance imaging (MRI) scanners operating at 3‐T and fitted with an eight‐channel receive‐only head coil (GE Signa System, General‐Electric) at the IoPPN, King's College, London, and Addenbrooke's Hospital, Cambridge. To ensure standardization of structural MRI scans across scanner platforms, a specialized acquisition protocol using quantitative imaging (driven equilibrium single‐pulse estimation of T1) was utilized. This protocol has previously been validated and extensively described elsewhere (Deoni et al., 2008; Ecker et al., 2012), resulting in high‐resolution structural T1‐weighted inversion‐recovery images, with 1x1x1mm resolution, a 256 × 256 × 176 matrix, TR = 1,800 ms, TI = 850 ms, FA = 20°, and FOV = 25.6 cm. DTI data were acquired using a spin‐echo echo‐planar imaging double refocused sequence providing whole head coverage with isotropic image resolution (2.4 × 2.4 × 2.4 mm; 32 diffusion‐weighted volumes with different noncollinear diffusion directions with b‐factor 1,300 s/mm^2^, and six nondiffusion‐weighted volumes; 60 slices without slice gap; TE = 104.5 ms; TR = 20 R‐R intervals; 128 × 128 acquisition matrix; FOV = 30.7 × 30.7 cm). The acquisition was gated to the cardiac cycle using a digital pulse oximeter placed on participants' forefinger.

### 2.3 Cortical reconstruction using FreeSurfer

We employed an automated analytical pipeline using FreeSurfer v6.0.0 software (http://surfer.nmr.mgh.harvard.edu/) to identify the GWM boundary by deriving models of the cortical surface for each T1‐weighted image. These well‐validated and fully automated procedures have been extensively described elsewhere (Dale, Fischl, & Sereno, 1999; Fischl & Dale, 2000; Fischl, Sereno, & Dale, 1999; Jovicich et al., 2006; Ségonne et al., 2004). In brief, a single‐filled white‐matter volume was generated for each hemisphere after intensity normalization, extra‐cerebral tissue was cropped, and image segmentation performed using a connected components algorithm. A triangular tessellated surface was then generated for each white‐matter volume. Deformation of this tessellated white matter surface resulted in a cortical mesh for the surfaces that defines the boundary between gray and white matter (i.e., white matter surface), and gray matter and cerebrospinal fluid (i.e., pial surface). This surface deformation is the result of the minimization of an energy functional that utilizes intensity gradients in order to place these surfaces where the greatest shift in intensity defines the transition between tissue classes (Dale et al., 1999). The use of intensity gradients across tissue classes assures that boundary placement is not reliant solely on absolute signal intensity and allows for subvoxel resolution in the placement of these boundary surfaces (Dale et al., 1999; Dale & Sereno, 1993; Fischl & Dale, 2000). These automated methods have previously been validated against histological analyses and have shown a high degree of accuracy in placing the GWM boundary (Rosas et al., 2002). The resulting surface models were visually inspected by three raters for reconstruction errors. Scans with segmentation errors were alternately manually edited by two of the raters and re‐assessed by all three, while scans of insufficient quality, mostly due to motion artifacts, were excluded from the analysis. For further details on quality assessments and the number of excluded scans, see Supplementary Methods 1.

### 2.4 Diffusion tensor MRI processing and coregistration with the structural MRI data

Diffusion data were first denoised and Gibb's ringing corrected using in‐house software (based on Kellner, Dhital, Kiselev, & Reisert, 2016; Veraart et al., 2016). Data outlier regeneration (*SD* = 4) and slice‐to‐volume correction (*SD* = 6) was performed using *eddy* from the FSL software toolbox (Andersson & Sotiropoulos, 2016). To maintain compatibility with previous studies (Catani et al., 2016), eddy current and motion correction, as well as robust tensor estimation (Tax, Jeurissen, Vos, Viergever, & Leemans, 2014) was performed using ExploreDTI (Leemans, Jeurissen, Sijbers, & Jones, 2009). To extract vertex‐wise parameter estimates for FA and MD sampled along the GWM boundary and at different sampling depths, we first coregistered each individual's diffusion data to the respective structural T1‐weighted image using the command *bbregister* in Freesurfer v6.0.0 (https://surfer.nmr.mgh.harvard.edu/fswiki/bbregister) (Greve & Fischl, 2009). Here, the FA mask in diffusion space was registered to the same‐subject anatomical image. The output of this transformation is a “register.dat” file which comprises the rigid‐body transformation matrix from the diffusion to the structural space. Finally, individual's diffusion data in structural space were registered to a common template, that is, fsaverage in Freesurfer, to allow for the calculation of between‐group differences.

### 2.5 Computation of diffusion measures at different sampling depths and of GWC

Diffusion measures, FA and MD, were sampled at each vertex along the GWM boundary (white matter surface), as well as at different percentile fractions of the total orthogonal distance projected from the white matter to pial surfaces (i.e., projection fractions) of 30% and 60% cortical thickness (CT) from the GWM boundary to the pial surface. These sampling depths within the gray matter were selected to capture diffusion at a deeper, and thus more heavily myelinated part of the gray matter (30% CT; Rowley et al., 2015; Sprooten et al., 2019), and at a more superficial, presumably lightly or not at all myelinated part of the gray matter (60% CT; Rowley et al., 2015; Sprooten et al., 2019). Further, within the white matter diffusion measures were sampled at absolute distances of −1 and −2 mm below the GWM boundary. These sampling depths were chosen to target the short‐range U‐fibers (at −1 mm) and to also capture the terminations of the long‐range fibers, which run between −1.5 and −2.5 mm below the GWM boundary (at −2 mm; Schüz & Braitenberg, 2002).

Based on previous work by our group (Andrews et al., 2017) and as adapted from Salat et al. (2009), the GWC was computed from the T1‐weighted images as the ratio between the gray matter signal intensity (GMI), sampled at a projection fraction of 30% CT into the cortical ribbon starting from the GWM boundary, in relation to the signal intensity of the white matter (WMI), sampled at an absolute distance of −1 mm into the white matter starting from the GWM boundary, at each cerebral vertex (*i*),

GWC_*i*_ = 100 × (WMI_*i*_ − GMI_*i*_)/ 0.5 × (WMI_*i*_ + GMI_*i*_).

Thus, a decreased GWC corresponds to a lower contrast between the intensities of gray matter at 30% CT and white matter at −1 mm and hence indicates a “blurring” of the GWM transition zone. To improve the ability to detect population changes, each parameter, that is, FA, MD, and GWC, was subsequently smoothed using a 15 mm full width at half maximum Gaussian kernel prior to statistical analyses.

### 2.6 Statistical analyses

Statistical analyses were conducted using the SurfStat toolbox (http://www.math.mcgill.ca/keith/surfstat/) for MATLAB (R2017b; MathWorks). Vertex‐wise statistical analyses of FA and MD measures sampled at the different sampling depths (i.e., −2 and −1 mm into the WM, at the GWM boundary, as well as 30 and 60% CT into the gray matter) as well as measures of GWC (Y) were estimated by the regression of a general linear model (GLM) with (a) diagnostic group, sex, and acquisition site as categorical fixed‐effects factors; (b) a group‐by‐sex interaction term; and (c) a linear and a quadratic age term, and FSIQ as continuous covariates:

*Y*_*i*_ = *β*_0_ *+ β*_1_Group + *β*_2_Sex + *β*_3_Group × Sex + *ß*_4_Site + *ß*_5_Age + *ß*_6_Age^2^ + *ß*_7_FSIQ + *ε*_*i*_,

where *ε*_*i*_ is the residual error at vertex *i*. Between‐group differences were estimated from the corresponding coefficient *β*_*1*_, normalized by the corresponding standard error. We further examined group‐by‐sex interaction effects (coefficient *β*_*3*_) across neuroanatomical features. All included continuous covariates were mean centered across groups to improve interpretability of the coefficients.

The utilized general linear model was derived after we initially assessed the goodness‐of‐fit of different model versions using nested model comparisons in a vertex‐wise fashion (see Supplementary Figures S1–S3). To determine if the addition of a new model term (e.g., group‐by‐sex interaction or the BDI total score) leads to a significantly improved goodness‐of‐fit, we employed a step‐up model selection procedure comparing the reduced model to the more complex model, and performed an *F*‐test for nested model comparisons at each vertex. This approach allows to identify the most parsimonious model at each vertex, that is, the most simple plausible model explaining the variability in the examined parameter of interest (i.e., FA, MD, or GWC) with the smallest set of predictors. All nested model comparisons were conducted in the total sample, that is, all individuals with ASD and controls. Here, the inclusion of the group‐by‐sex interaction term yielded an improved goodness‐of‐fit, whereas the additional inclusion of the BDI was not significant (see Supplementary Figures S1–S3). The group‐by‐sex interaction term was therefore included as a model term in all subsequent analyses.

As previous findings by our group indicate the developmental trajectory of GWC to incorporate linear as well as nonlinear effects of age in ASD (Mann et al., 2018), we further included a linear as well as a quadratic age term in our GLM to control for those and possible age‐related between‐group differences in FA and MD. The developmental trajectory of FA and MD is also expected to differ between groups. In neurotypical development, the myelination process follows a quadratic trajectory over the lifespan (Bartzokis, 2004a, 2004b; Bartzokis et al., 2001, 2003; Sowell et al., 2003; see also Rowley et al., 2017 for intracortical myelin and Ouyang, Kang, Detre, Roberts, & Huang, 2017 for the superficial white matter).

To control for the presence of comorbid depressive symptoms, we repeated the analyses following the exclusion of individuals with antidepressant medication intake and/or a comorbid diagnosis of depression. In addition, to test the robustness of our findings, we further repeated the analyses when covarying for depressive symptoms, as assessed by BDI. Moreover, to control for variability in total gray and white matter volumes, we examined the main effect of group when covarying for the effect of total brain measures. Finally, we repeated the analyses when stratifying our sample by sex.

In all surface‐based analyses, corrections for multiple comparisons across the whole brain were performed using random field theory‐based cluster analysis for nonisotropic images using a cluster‐based significance threshold of *p* < .05 (two‐tailed; Worsley, Andermann, Koulis, MacDonald, & Evans, 1999). To determine the functional relevance of the observed findings, we further calculated Pearson correlation coefficients between scores in clinical questionnaires assessing ASD‐related clinical symptomatology and common comorbidities (i.e., anxiety, depression) with the mean FA, MD, and GWC extracted from clusters obtained in the main effect of group analyses.

To identify the extent to which the spatially distributed patterns of significant between‐group differences of the different parameters, GWC, as well as FA and MD sampled at the GWM boundary, were either unique to one measure or overlapping, we further conducted pair‐wise *χ* ^2^ tests, to test the null hypotheses that differences in either two parameters (GWC and FA, GWC and MD, as well as FA and MD) are equally distributed. Moreover, to examine if the respective observed degree of overlap supports the assumption of two spatially (in)dependent patterns, we utilized a simulation strategy generating *N* = 5,000 random difference maps, that incorporated random *t*‐values that were thresholded at *p* < 0.05 for the respective measures (i.e., GWC, FA, and/or MD). The degree of overlap, estimated as number of vertices with alterations in the respective two measures, was subsequently calculated in each simulation. This provides a probability estimation of obtaining the observed percentage of overlap based on randomly varying difference patterns.

## 3 RESULTS

### 3.1 Participant demographics and global brain measures

There were no significant differences between individuals with ASD and TD controls in age (*t*(181.12) = 1.62, *p* = .11), FSIQ (*t*[174.12) = 1.69, *p* = .09), or total brain measures, such as total brain volume (*t*(178.58) = −0.81, *p* = .42), total surface area (*t*(177.42) = −0.87, *p* = .38), and mean CT (*t*(181.06) = −0.51, *p* = .61; see Table 1). For a detailed clinical characterization of the sample, see Supplementary Table S1.

### 3.2 Between‐group differences in FA at the different sampling depths

**FIGURE 1:**
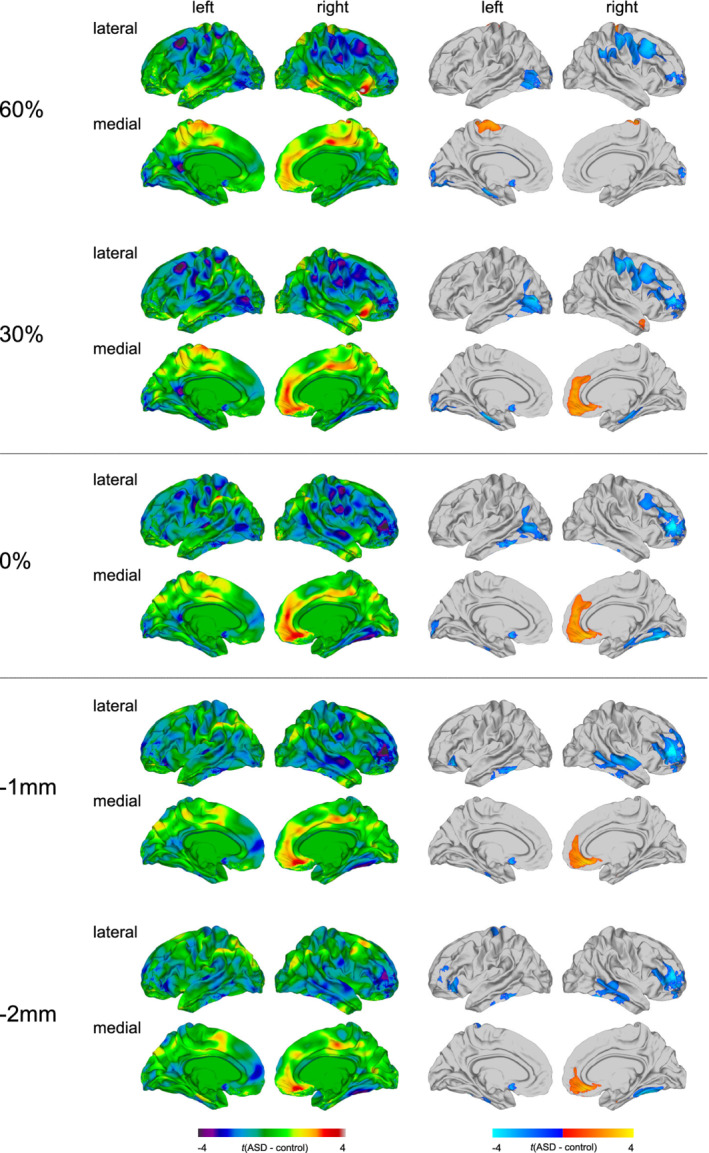
Main effect of group for fractional anisotropy (FA). Regions of increased and decreased FA in individuals with autism spectrum disorder (ASD) compared to typically developing (TD) controls at the gray‐white matter (GWM) boundary (0%), at different cortical thickness (CT) projection fractions within the gray matter (i.e., 30 and 60% CT, sampled from the GWM boundary into the thickness of the cortical ribbon), and within the superficial white matter (sampled at absolute distances of −1 and −2 mm below the GWM boundary). Displayed are the unthresholded (left panel) and thresholded (right panel) *t*‐maps, where increased FA estimates in ASD are marked in yellow to red (left panel), respectively, red to yellow (right panel), and decreased FA estimates in ASD are marked in cyan to purple (left panel), respectively, blue to cyan (right panel; random field theory [RFT]‐based cluster corrected *p* < .05, two‐tailed)

Individuals with ASD relative to TD controls predominantly displayed significant reductions in FA, which were most extensive at the GWM boundary, and gradually decreased in spatial extent with increasing sampling distance from the GWM boundary (see Figure 1 and Supplementary Table S2). More specifically, significantly reduced FA in ASD was observed across sampling depths in the right prefrontal cortex (Brodmann area [BA] 10/45–46), as well as the left fusiform and inferior temporal gyrus (BA 20/37). Within the superficial white matter, reduced FA was further observed in the right temporal lobe (BA 20–22) and in the left lateral orbitofrontal cortex (BA 10/45/47). At the GWM boundary and within the gray matter, reduced FA was further observed in right frontoparietal (BA 1–4/6/39–40) and left occipitotemporal regions (BA 19/37). In contrast, significant increases in FA in ASD were observed across sampling depths in a large cluster centered on the right medial orbitofrontal cortex and superior frontal gyrus (BA 9–11/32). At 60% CT exclusively, FA was significantly increased in two small clusters located on the right precentral and postcentral gyrus (BA 1–4), as well as in the left paracentral lobule (BA 4). The results pattern remained largely unchanged upon exclusion of individuals with antidepressant medication intake and/or a comorbid diagnosis of depression (see Supplementary Figure S4), as well as when covarying for BDI total score or total gray and white matter volumes, respectively (see Supplementary Figures S5a and S6). The results for the analysis conducted with males and females separately are presented in Supplementary Figures S7 and S8.

### 3.3 Between‐group differences in MD at the different sampling depths

**FIGURE 2:**
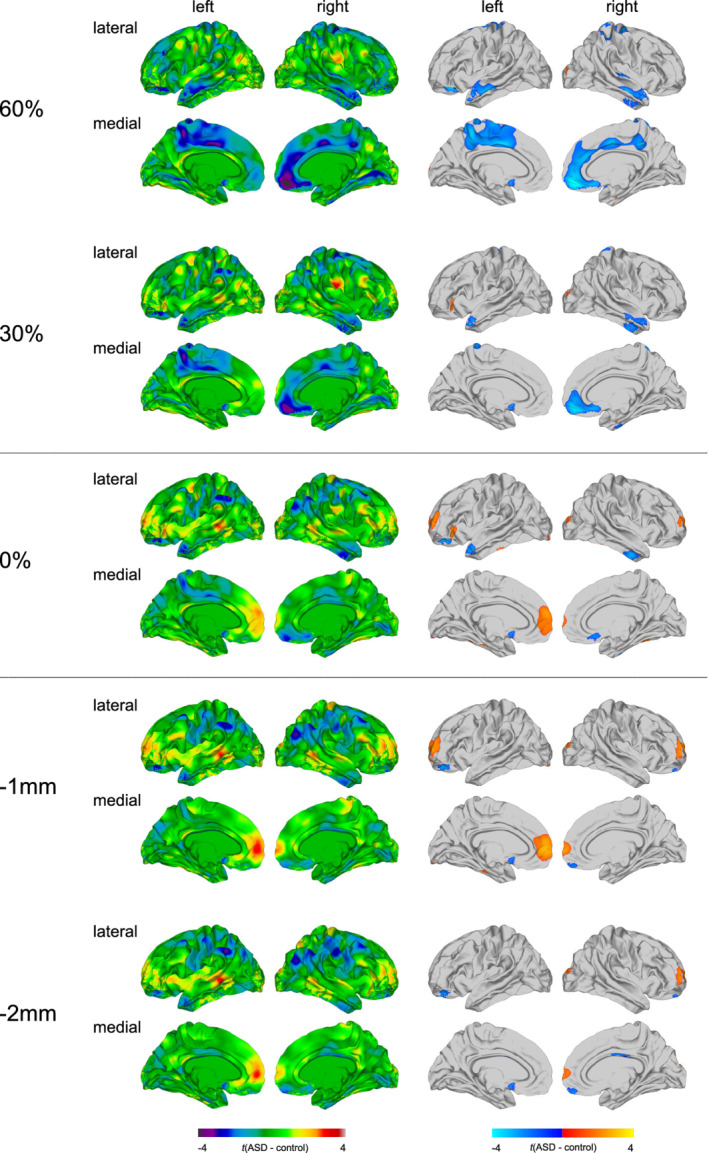
Main effect of group for mean diffusivity (MD). Regions of increased and decreased MD in individuals with autism spectrum disorder (ASD) compared to typically developing (TD) controls at the gray‐white matter (GWM) boundary (0%), at different cortical thickness (CT) projection fractions within the gray matter (i.e., 30 and 60% CT, sampled from the GWM boundary into the thickness of the cortical ribbon), and within the superficial white matter (sampled at absolute distances of −1 and −2 mm below the GWM boundary). Displayed are the unthresholded (left panel) and thresholded (right panel) *t*‐maps, where increased MD estimates in ASD are marked in yellow to red (left panel), respectively, red to yellow (right panel), and decreased MD estimates in ASD are marked in cyan to purple (left panel), respectively, blue to cyan (right panel; random field theory [RFT]‐based cluster corrected *p* < .05, two‐tailed)

At the GWM boundary and within the superficial white matter, individuals with ASD mostly had significantly increased measures of MD relative to TD controls. These increases were predominantly observed in bilateral frontal lobes (BA 10–11/45), including the orbitofrontal cortex and pars triangularis, as well as in the left fusiform and inferior temporal gyrus (BA 20/37; see Figure 2 and Supplementary Table S3). In contrast, we observed widespread MD reductions in ASD within the cortical gray matter. Those were most extensive at 60% CT and gradually increased in spatial extent with increasing CT projection fraction. More specifically, individuals with ASD had significantly decreased MD in the bilateral temporal lobes (BA 20–22), pre‐ and postcentral gyrus (BA 1–4), as well as in a large cluster in the right medial orbitofrontal cortex and superior frontal gyrus (BA 9–11/32). At 60% CT only, decreases in MD were also observed in a large cluster centering on the left paracentral lobule (BA 4/6–7/24/31). Neither the exclusion of individuals with antidepressant medication intake and/or a comorbid diagnosis of depression (see Supplementary Figure S9), nor the inclusion of the BDI total score or total gray and white matter volumes as covariate/s resulted in a significant change of the resulting patterns of between‐group differences (see Supplementary Figures S5b and S10). The results stratified by sex are presented in Supplementary Figures S11 and S12.

### 3.4 Between‐group differences in GWC

**FIGURE 3:**
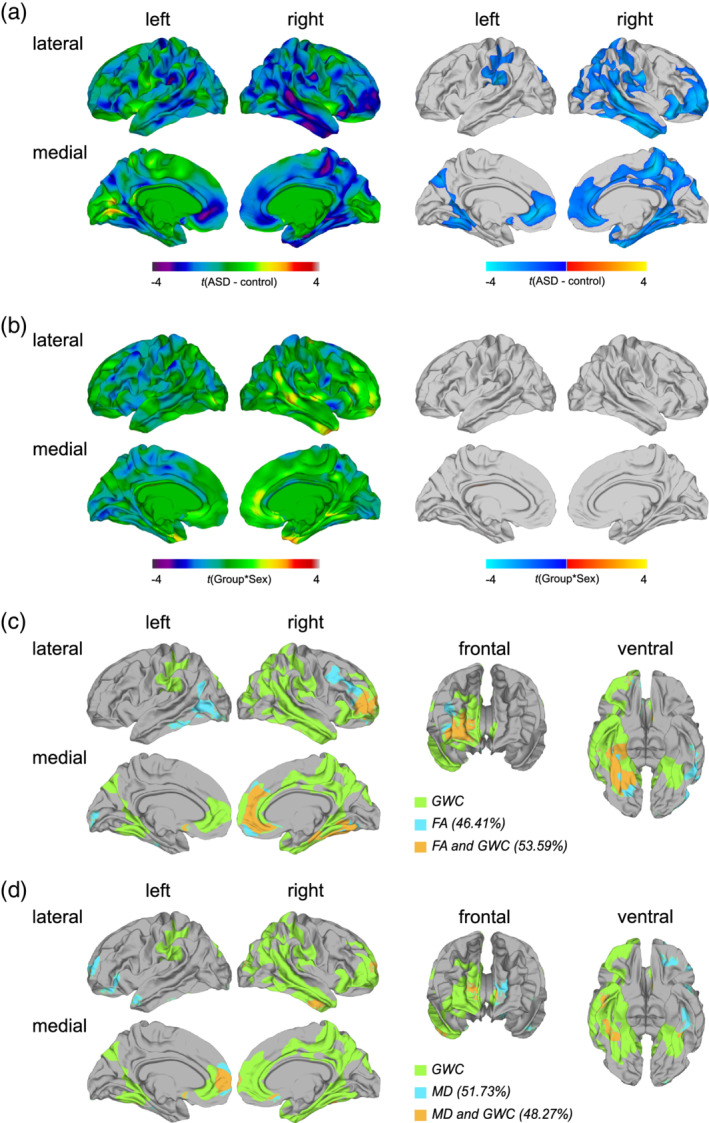
Main effect of group and group‐by‐sex interaction effects for the gray‐white matter tissue contrast (GWC), and the overlap between the patterns of between‐group differences in GWC and diffusion measures. (a) Regions of significantly reduced GWC in individuals with autism spectrum disorder (ASD) compared to typically developing (TD) controls sampled as ratio between the gray matter signal intensity sampled 30% into the thickness of the cortical ribbon from the gray‐white matter (GWM) boundary and white matter signal intensity sampled −1 mm below the GWM boundary (random field theory [RFT]‐based cluster corrected *p* < .05, two‐tailed). (b) Regions with a significant group‐by‐sex interaction effect for GWC. Displayed are the unthresholded (left panel) and thresholded (right panel) *t*‐maps (RFT‐based cluster corrected *p* < .05, two‐tailed). (c) As well as percentages of spatial overlap between the patterns of between‐group differences in GWC with fractional anisotropy (FA) and (d) with mean diffusivity (MD), both sampled vertex‐wise at the GWM boundary

As reported previously (Andrews et al., 2017), the GWC was significantly reduced in individuals with ASD in several clusters across the cortex (see Figure 3a and Supplementary Table S4). These reductions were observed bilaterally in the fusiform gyrus (BA 37), the postcentral gyrus (BA 1–3), the inferior and superior parietal cortex (BA 7/40), the occipital cortex (BA 18–19), the medial orbitofrontal cortex (BA 10–11), and the cingulate cortex (BA 24/30/32–33). Overall, the right hemisphere was more affected than the left, with reductions in GWC in ASD expanding to the precentral gyrus and paracentral lobule (BA 4), the inferior, middle, and superior temporal gyri and temporal pole (BA 20–21/38/41), the posterior cingulate cortex (BA 23/31), as well as the ventrolateral and dorsolateral prefrontal cortex (BA 9/44–46). There were no clusters where individuals with ASD showed a significant increase in GWC as compared to TD controls. The results pattern remained largely unchanged when individuals with antidepressant medication intake and/or a comorbid diagnosis of depression where excluded from the analysis (see Supplementary Figure S13), as well as when BDI total score or total gray and white matter volumes were included as covariate/s (see Supplementary Figures S5c and S14). For the results stratified by sex, see Supplementary Figure S15. We did not, however, observe any significant group‐by‐sex interaction effects for measures of GWC (see Figure 3b).

### 3.5 Group‐by‐sex interaction effects in FA at the different sampling depths

**FIGURE 4:**
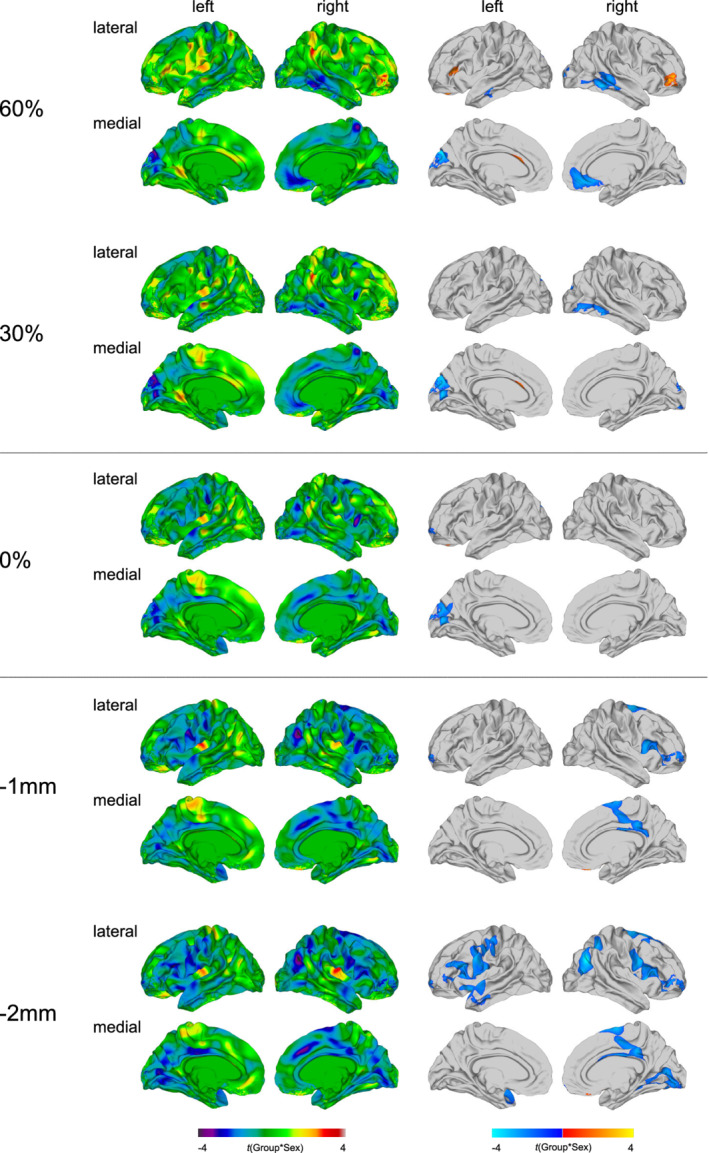
Group‐by‐sex interaction effects for fractional anisotropy (FA). Regions with a significant group‐by‐sex interaction effect for FA at the gray‐white matter (GWM) boundary (0%), at different cortical thickness (CT) projection fractions within the gray matter (i.e., 30 and 60% CT, sampled from the GWM boundary into the thickness of the cortical ribbon), and within the superficial white matter (sampled at absolute distances of −1 and −2 mm below the GWM boundary). Displayed are the unthresholded (left panel) and thresholded (right panel) *t*‐maps (random field theory [RFT]‐based cluster corrected *p* < .05, two‐tailed)

In addition to the main effect of group, we observed significant group‐by‐sex interactions for measures of FA (see Figure 4 and Supplementary Table S5). These were most pronounced within the superficial white matter and increased in spatial extent with increasing sampling distance away from the GWM boundary. Significant group‐by‐sex interactions within the superficial white matter were located in right paracentral and cingulate regions (BA 4/23–24/30–31), as well as in the right orbitofrontal and ventrolateral prefrontal cortex (BA 10/44–45). At −2 mm within the white matter, significant group‐by‐sex interaction effects were further observed in the left ventrolateral and dorsolateral prefrontal cortex (BA 44–46), the bilateral supplementary motor area (SMA; BA 6), the bilateral precentral and postcentral gyri (BA 1–4), the left insula (BA 13), the left middle and superior temporal gyrus (BA 21–22), the right superior frontal gyrus (BA 8), as well as the right inferior parietal cortex (BA 39).

At the GWM boundary and within the cortical gray matter, group‐by‐sex interactions in FA were observed in the left cuneus and pericalcarine cortex (BA 18–19). At 30% CT, significant group‐by‐sex interactions were further observed in right temporo‐occipital regions (BA 18–19/37), whereas at 60% CT, significant group‐by‐sex interactions were additionally located in the left pars triangularis (BA 45), the left middle temporal gyrus (BA 20–21), as well as right medial orbitofrontal, cingulate, and temporal regions (BA 11/20–22/24/32). The significant interaction in the left pars triangularis at 60% CT was the only cluster, in which mean FA was decreased in males relative to females in controls, while in individuals with ASD females had lower FA values than males. In all other significant clusters mean FA was increased in neurotypical males relative to neurotypical females, while in the ASD group, females had higher FA values compared to males (for boxplots see Supplementary Figure S16).

### 3.6 Group‐by‐sex interaction effects in MD at the different sampling depths

**FIGURE 5:**
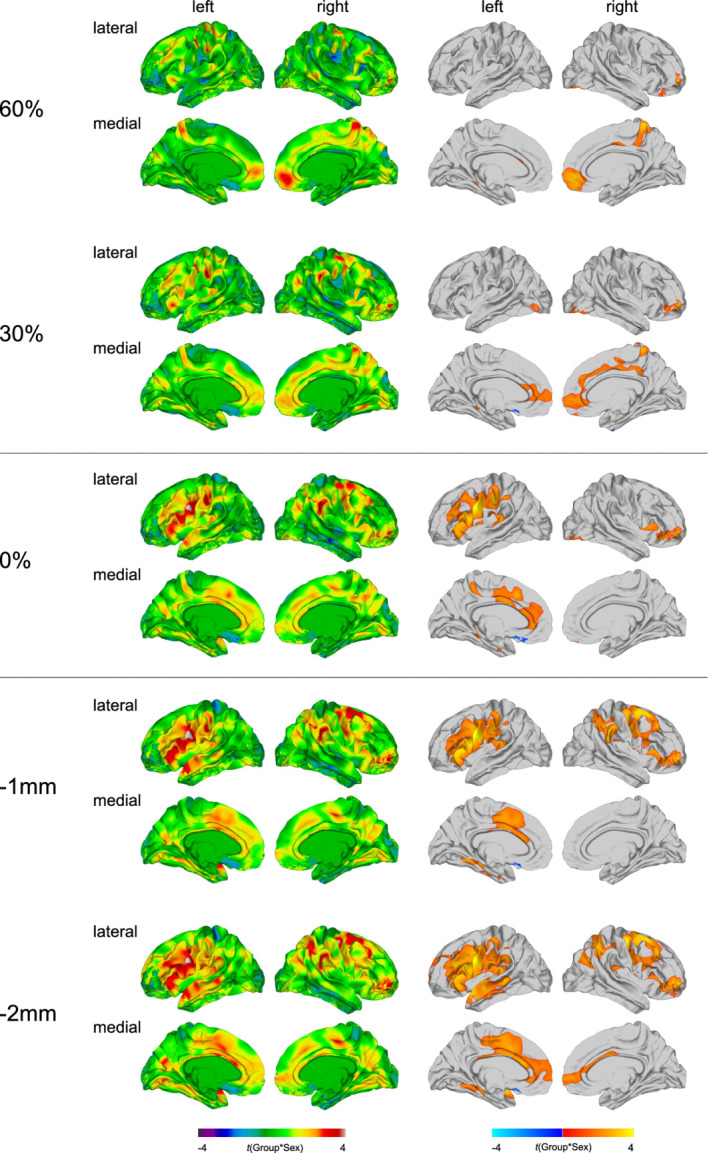
Group‐by‐sex interaction effects for mean diffusivity (MD). Regions with a significant group‐by‐sex interaction effect for MD at the gray‐white matter (GWM) boundary (0%), at different cortical thickness (CT) projection fractions within the gray matter (i.e., 30 and 60% CT, sampled from the GWM boundary into the thickness of the cortical ribbon), and within the superficial white matter (sampled at absolute distances of −1 and −2 mm below the GWM boundary). Displayed are the unthresholded (left panel) and thresholded (right panel) *t*‐maps (random field theory [RFT]‐based cluster corrected *p* < .05, two‐tailed)

Significant group‐by‐sex interactions for MD were most distinctive within the superficial white matter, particularly at the deeper sampling depth of −2 mm (see Figure 5 and Supplementary Table S6). At the GWM boundary and within the superficial white matter, significant group‐by‐sex interactions were observed in left cingulate regions (23–24/31–33), left fronto‐central and fronto‐parietal regions (BA 1–4/6/40), including the pre‐ and postcentral gyrus and the supramarginal gyrus, as well as bilateral orbitofrontal, ventrolateral, and dorsolateral prefrontal regions (BA 10–11/44–47). Within the superficial white matter, particularly at −2 mm, significant group‐by‐sex interactions for MD were further observed in the left temporal lobe (BA 20–22/37–38/41), as well as the right precentral and postcentral gyrus (BA 1–4), the right middle and superior frontal gyrus (BA 4/6/8–9), and the right inferior and superior parietal cortex (BA 7/39–40).

Within the cortical gray matter, significant group‐by‐sex interactions for MD were located in the right medial orbitofrontal and superior frontal gyrus (BA 10–11), the right anterior and posterior cingulate cortex (BA 23–24/31–32), the right paracentral lobule (BA 4), and the right precuneus cortex (BA 7). At 30% CT, a significant group‐by‐sex interaction was additionally observed in the left anterior prefrontal and cingulate cortex (BA 10/24/32–33). In these clusters, mean MD was decreased in neurotypical males relative to neurotypical females, while in the ASD group, mean MD was equal or slightly higher in males as compared to females (for boxplots see Supplementary Figure S17).

### 3.7 Overlap of between‐group differences in FA and MD, both sampled at the GWM boundary, with between‐group differences in GWC

Across hemispheres and out of the total number of vertices (*n* = 327,684), the number of vertices with a significant between‐group difference in GWC (*n* = 88,131) significantly exceeded the number of vertices with atypical FA (*n* = 17,046; *χ* ^2^(*df* = 1) = 57,226, *p* < .001) and altered MD (*n* = 6,849; *χ* ^2^(*df* = 1) = 81,347, *p* < .001) in ASD. Furthermore, out of all vertices with a significant between‐group difference in FA, *n* = 9,135 vertices (i.e., 53.59%) also displayed a significant alteration in GWC (see Figure 3c). Similar proportions of overlap were observed when examining clusters with significant between‐group differences in MD, where 48.27% (i.e., *n* = 3,306) of vertices with a difference in MD overlapped with vertices with significantly altered GWC in ASD (see Figure 3d). Simulations revealed that the probability of obtaining the same degree of overlap (i.e., 53.59 or 48.27%, respectively) or higher by chance is less than *p* < .001 for both overlap patterns. Moreover, the number of vertices with a significant between‐group difference in FA (*n* = 17,046) significantly exceeded the number of vertices with altered MD (*n* = 6,849; *χ* ^2^(*df* = 1) = 4,515, *p* < .001) sampled at the GWM boundary. The degree of spatial overlap between these patterns of between‐group differences in FA and MD was very low with only *n* = 776 vertices (i.e., 4.55%) displaying a significant alteration in both features.

### 3.8 Associations between atypical diffusion at the GWM boundary and reduced GWC with clinical symptom severity

We observed significant correlations between the severity of clinical symptoms with some of the clusters identified by the main effect of group analyses (see Supplementary Figure S18). As expected, the strongest associations between clinical measures and neuroanatomical features were observed with measures of core ASD symptom severity (e.g., assessed using the AQ total score), with significant negative correlations with measures of FA and GWC, and significant positive correlations with measures of MD. This indicates that more pronounced ASD symptoms are associated with a more distinctive “blurring” of the GWM boundary, as well as more pronounced decreases in FA and increases in MD at the GWM boundary. Notably, this association was stronger for measures of GWC and MD as compared to FA, which we observed to be more strongly associated with comorbid symptoms of depression and anxiety (i.e., BDI and BAI scores).

## 4 DISCUSSION

We aimed to examine neuroanatomical differences at and around the GWM boundary in ASD individuals relative to TD controls based on measures of diffusion, and to determine if alterations are modulated by sex and dependent on the examined tissue class (i.e., gray or white matter) and cortical depth. Moreover, we aimed to establish how variability in measures of diffusion sampled at the GWM boundary relates to regional differences in GWC in ASD. We observed that diffusion properties at the GWM boundary and sampled within the adjacent gray and superficial white matter are significantly altered in ASD, with between‐group differences and group‐by‐sex interactions depending on tissue class and gray and white matter sampling depths. Furthermore, the regionally distributed pattern of differences in diffusion parameters partially and nonrandomly overlapped with between‐group differences in GWC. Hence, we replicate and extend the results of former studies; and taken together highlight the GWM boundary as a pivotal location of neuroanatomical abnormality in ASD in adulthood, which is modulated by sex. This may help guide future genetic and/or histological studies into the etiology and brain development related to the condition.

Converging lines of evidence coming from histological, genetic, and in vivo neuroimaging studies indicate that neuroanatomical alterations, specifically around the GWM boundary, constitute a crucial aspect of the pathophysiology of ASD (Andrews et al., 2017; Avino & Hutsler, 2010; Hoerder‐Suabedissen et al., 2013). However, the GWM boundary in ASD had not yet been characterized based on measures of diffusion as assessed using DTI, which have been linked to several aspects of underlying white matter neuroarchitecture including myelination and fiber architecture (Beaulieu, 2002; Jbabdi et al., 2015; Jones et al., 2013). Here, we established that measures of FA and MD are significantly altered at the GWM boundary in ASD as compared to TD controls predominantly in (a) fronto‐parietal, prefrontal, and temporo‐occipital regions for measures of FA, and (b) in prefrontal regions and in the fusiform gyrus for measures of MD. Neuroanatomical abnormalities in these regions have previously been associated with ASD symptoms that include difficulties in the processing of faces and biological motion and impairments on theory of mind tasks and various aspects of social cognition (for reviews, see Amaral, Schumann, & Nordahl, 2008; Ecker, 2017).

Moreover, in similar regions, we previously reported abnormalities in the GWC in ASD (Andrews et al., 2017). The current study was also motivated by histological reports of a more “indistinct” GWM transition zone in ASD (Avino & Hutsler, 2010). Here, we demonstrate that ASD‐related neuroanatomical variation in diffusion characteristics sampled at the GWM boundary overlapped to a large degree (about 50%) with differences in GWC. Simulations revealed that the degree of spatial overlap between the patterns for FA with GWC, and MD with GWC, are highly statistically significant (i.e., beyond what would be predicted by chance). Similar to measures of diffusion, the GWC has also been related to myelin content as it is based on differences in signal intensity derived from the T1‐weighted image, which reflects cholesterol, a major constitute of myelin (Koenig, 1991; Koenig, Brown, Spiller, & Lundbom, 1990). However, measures of diffusion also depend on other aspects of neural architecture, such as fiber density or alterations in neuropil (i.e., Beaulieu, 2002; Selemon & Goldman‐Rakic, 1999). This might explain that while between‐group differences in both features significantly overlap in about 50% of vertices, the rest (i.e., 50%) of alterations in FA and MD at the GWM boundary remain unexplained by variability in GWC. Thus, even though there is a high correlation between diffusion metrics and interindividual variability in tissue contrast, both of these features capture neuroanatomical information that is unique, and that may reflect distinct aspects of the neural architecture.

Beyond characterization of the GWM boundary in ASD based on measures of diffusion, we also aimed to establish if diffusion metrics are significantly altered at different sampling depths within the superficial white matter and gray matter. To date only one study has examined atypicalities in the neuroarchitecture of the superficial white matter in ASD using DTI, where diffusion metrics were sampled at −2 mm below the GWM boundary (Hong et al., 2019). In this study, the authors reported decreased FA and increased MD in ASD predominantly in medial parietal and temporoparietal regions. These previous results correspond with the findings presented in our study, where we also observed reductions in FA and increases in MD sampled at −1 and −2 mm below the GWM boundary. However, compared to the work by Hong et al. (2019), we report that these differences were most pronounced in prefrontal and temporal regions, including the inferior temporal and fusiform gyrus, rather than parietal areas. Our results thus partially differ in terms of the spatial distribution, which is likely attributable to methodological differences such as surface generation and the different age range investigated across studies. Nonetheless, taken together, there is growing evidence to suggest that the integrity of the superficial white matter is significantly altered in ASD, and that this may affect measures of cortico‐cortical structural and functional connectivity (Hong et al., 2019; Shukla et al., 2011; Sundaram et al., 2008). In addition, our results correspond with previous findings suggesting that between‐group differences in diffusion measures within the white matter in ASD are modulated by sex (Andrews et al., 2019; Beacher et al., 2012; Irimia, Torgerson, Jacokes, & Van Horn, 2017; Lei et al., 2019; Nordahl et al., 2015; Zeestraten et al., 2017). Our findings further show that this sexdependency of diffusion alterations in ASD extends to measures of diffusion sampled at the GWM boundary and within the cortical gray matter, however, is most prominent within the white matter tissue compartment. Moreover, our findings expand on previous reports by suggesting that alterations in the superficial white matter in ASD are depth‐dependent, with between‐group differences being more prominent in closer proximity to the GWM boundary, whereas group‐by‐sex interaction effects were most pronounced at a deeper sampling depth (i.e., −2 mm).

We also observed significant differences in both FA and MD sampled within the cortical gray matter. However, the magnitude and sign of the difference varied depending on the particular sampling depth (i.e., at 30 and 60% CT). For example, the increase in FA in the right medial prefrontal cortex in ASD was only observed at 30% CT projection fraction (and lower), but not at 60% CT. In contrast, the decrease in MD in this brain region was most pronounced at 60% CT, but was not observed at or beneath the GWM boundary. The observed decreases in MD within the gray matter was an unexpected finding, indicating diffusion to be more restricted in ASD relative to TD controls—which might indicate an increase in the number or density of cells. This finding is in contrast to the observed decreases in FA within the gray matter and the commonly reported accelerated cortical thinning and resultant decreased CT in ASD during adulthood (i.e., Wallace, Dankner, Kenworthy, Giedd, & Martin, 2010; Zielinski et al., 2014), which both suggest a reduction in the number and/or density of cells. However, the age‐related trajectory of CT deviations in ASD seems to be region‐specific. For instance, in some frontal and temporal regions decelerated cortical thinning during early adulthood up to middle age has been observed, leading to region‐wise increases in CT in ASD (Raznahan et al., 2010; Zielinski et al., 2014). Hence, the observed regional decreases in MD might be linked to regional increases in CT, and indicate an increase in the number or density of cells within those regions in ASD.

Similarly, the differential results we obtained with regard to variation in sampling depths within the cortical gray matter might partially be explained by its layer‐specific neuroarchitectural make‐up. For example, the deeper layers of the cortex (IV–VI) are typically more myelinated (Nieuwenhuys, 2013), and will thus have an MRI signal that is different from the signal in the superficial gray matter. In fact, it is estimated that the myelinated part of the cortical gray matter can extend up to 70% CT, particularly in higher association areas, such as somatosensory, motor, auditory, and visual cortices (Rowley et al., 2015). In most other brain regions, however, the proportion of the myelinated to the unmyelinated gray matter comprises approximately 30–40% (Rowley et al., 2015). The variability of density and orientation of myelinated axons across cortical layers is hence also expected to affect measures of diffusion at different sampling depths. Based on our finding of a more pronounced between‐group difference in MD at 60% as compared to 30%, it seems that diffusion properties might be more affected in unmyelinated relative to myelinated gray matter; and this might be an important venue for future histopathological research.

Moreover, our results within the cortical gray matter suggest that FA and MD might capture different aspects of the neural architecture that could explain their layer‐specificity, that is, more superficial layers showing a more significant discrepancy in MD compared to FA between groups. This is further supported by the observation that the spatial overlap of between‐group differences in FA and MD at the GWM boundary is only ~5%, and that both of these features show differential neurodevelopmental trajectories in TD individuals (Lebel et al., 2012). Consistently, in the present study, alterations in these parameters at the GWM boundary in ASD were associated with distinct clinical features, with atypical MD being associated with the severity of core ASD symptomatology, whereas altered FA showed a more pronounced association with the severity of comorbid symptoms of anxiety and depression. As the severity of comorbid symptoms was strongly correlated with the severity of ASD symptoms, between‐group differences in FA might hence be more reflective of the overall “symptom load” (i.e., across conditions), while brain‐based alterations in MD and GWC seem to be more sensitive to variability in core ASD symptoms. The high intercorrelations between questionnaire scores further indicate that individuals who are more severely affected in terms of autism symptoms also seem to experience more pronounced symptoms in comorbid conditions, such as depression and anxiety, which seems to translate to the level of neuroanatomy.

Our results need to be interpreted in the light of several methodological considerations. First, to overcome single‐site recruitment limitations we utilized a multi‐center design. However, provided that MRI hardware and data processing factors are controlled for, measures of surface anatomy generated by FreeSurfer have been demonstrated to be highly reliable across field‐strengths and scanner platforms (Han et al., 2006). Furthermore, the same stringent quality assessment and preprocessing pipeline was applied to all surface reconstructions, and intersite effects were accounted for in the statistical model. Second, partial volume effects may affect our estimates of GWC and diffusion metrics. However, their impact on both groups is expected to be equal and the observed significant between‐group differences and group‐by‐sex interactions are thus not expected to be attributable to this. Third, the derived estimates of diffusion properties and tissue intensities, used to calculate the GWC, remain dependent on the current resolution of diffusion and structural MRI images (i.e., 2.4 and 1 mm isotropic voxels, respectively). This resolution does not allow the delineation of the distinct cortical layers identified by histology. To allocate the identified depth‐dependent neuroanatomical variation in ASD to discrete cortical layers, images of higher spatial resolution are required. Finally, within the scope of this study, we did not examine associations between the observed diffusion alterations at and around the GWM boundary and intellectual impairment, as all our included participants had an IQ above 70. Our sample is therefore not representative of all individuals on the autism spectrum, and future research is needed to elucidate the relevance of these neuroanatomical findings on adaptive functioning in individuals with ASD.

In conclusion, this is the first study to provide evidence of neuroanatomical variations at the GWM boundary, as estimated by diffusion metrics, in adult ASD individuals compared to TD controls. In accordance with previous findings from postmortem, genetic, and structural neuroimaging studies, our results provide further support for the notion of the GWM boundary as a crucial location of neuroanatomical abnormality in ASD. Moreover, our findings suggest that atypical measures of diffusion in ASD are modulated by sex and not only depend on cortical region and tissue class (i.e., gray or white matter) but also on the sampling depth within the respective tissue.

## CONFLICT OF INTERESTS

Prof E. T. B. is employed half‐time by GlaxoSmithKline and holds GSK shares. None of the remaining authors have declared any conflict of interest or financial interests, which may arise from being named as an author on the manuscript.

## Supporting information


**Appendix** **S1**: Supporting Information

## Data Availability

The full set of raw data is not currently publicly available due to ethical restrictions. However, a subset of the sample can be made available upon request.
