# Supplementary material for: Atypical measures of diffusion at the gray‐white matter boundary in autism spectrum disorder in adulthood
Source: Hum Brain Mapp. 2020 Oct 23;42(2):467–84. doi: 10.1002/hbm.25237 (PMC7775996; doi:10.1002/hbm.25237)
Supplement: Supplementary file 1 — Appendix S1: Supporting Information [file HBM-42-467-s001.pdf]

Supplementary Materials

Supplementary Methods *2*

1. Quality Assessment 2

Supplementary Tables *3*

Supplementary Table S1 3

Supplementary Table S2 4

Supplementary Table S3 6

Supplementary Table S4 8

Supplementary Table S5 9

Supplementary Table S6 10

Supplementary Figures *12*

Supplementary Figure S1 12

Supplementary Figure S2 12

Supplementary Figure S3 12

Supplementary Figure S4 13

Supplementary Figure S5 14

Supplementary Figure S6 15

Supplementary Figure S7 16

Supplementary Figure S8 17

Supplementary Figure S9 18

Supplementary Figure S10 19

Supplementary Figure S11 20

Supplementary Figure S12 21

Supplementary Figure S13 22

Supplementary Figure S14 23

Supplementary Figure S15 24

Supplementary Figure S16 25

Supplementary Figure S17 26

Supplementary Figure S18 27

References *28*

Supplementary Methods

1. Quality Assessment

Initially, three raters blind to the diagnosis visually inspected the image quality for the surface models derived by FreeSurfer v6.0.0 of 266 structural T1-weighted images. The image quality of the scans was rated on the basis of the following three categories: (1) good quality, i.e., no visible reconstruction errors or artifacts; scans of this category were accepted as is and no manual edits were performed; (2) with visible reconstruction errors in pial and/or white matter surface; for scans in this category manual edits were performed and scans were subsequently re-preprocessed and re-assessed (n=78, i.e., 29% of the total sample; out of which n=28 did not improve after manual editing and were thus excluded); or (3) with gross anatomical abnormalities or severe acquisition or motion artifacts; scans of this category were excluded prior to the analysis (n=15, i.e., 6% of the total sample). Based on this quality assessment, the quality of the Diffusion Tensor Imaging (DTI) data of the remaining n=223 participants was visually inspected with all participants with a T1-weighted image of good quality also having a good quality DTI scan. Manual edits were only performed in regard to the T1-weighted images, as eddy current and motion correction of the DTI scans was already implemented in the framework of the preprocessing pipeline. After the quality assessments, we further excluded 39 participants in order to obtain two groups that were matched on age and full-scale IQ. This led to a final sample size of N=184 participants.

Supplementary Tables

Supplementary Table S1. Clinical Characterization

	ASD	TDC	Test Statistic	Significance
AQ[a]	33.92 ± 8.37 (12–48)	13.46 ± 6.18 (1–32)	t=18.56	p<0.001
EQ[b]	25.25 ± 16.68 (3–82)	47.49 ± 12.61 (13–75)	t=−9.98	p<0.001
SQ[c]	67.34 ± 25.38 (20–139)	55.46 ± 19.16 (16–96)	t=3.51	p<0.001
BDI[d]	13.41 ± 9.71 (0–41)	5.43 ± 5.28 (0–17)	t=6.17	p<0.001
BAI[b]	14.42 ± 10.76 (0–48)	5.63 ± 6.50 (0–31)	t=6.57	p<0.001
OCI–R[e]	16.51 ± 13.45 (1–65)	16.86 ± 13.59 (0–59)	t=−0.17	p=0.86

Note: Data expressed as mean ± *SD* (range). Data displayed is based on raw scores. Abbreviations: ASD: autism spectrum disorder; TDC: typically developing controls; AQ: Autism Spectrum Quotient (Baron–Cohen et al., 2001); EQ: Empathy Quotient (Baron–Cohen & Wheelwright, 2004); SQ: Systemizing Quotient (Baron–Cohen et al., 2003); BDI: Beck Depression Inventory (Beck & Steer, 1987); BAI: Beck Anxiety Inventory (Beck & Steer, 1993); OCI–R: Obsessive–Compulsive Inventory–Revised (Abramowitz & Deacon, 2006; Huppert et al., 2007); [a]based on N=178 (n=89 ASD, n=89 TDC); [b]based on N=176 (n=88 ASD, n=88 TDC); [c]based on N=176 (n=89 ASD, n=87 TDC); [d]based on N=177 (n=88 ASD, n=89 TDC); [e]based on N=179 (n=88 ASD, n=91 TDC).

Supplementary Table S2. Clusters with significantly increased and decreased fractional anisotropy (FA) at the gray-white matter boundary (0%) and different sampling depths within the gray matter and superficial white matter

Sampling Depth	Contrast	Cluster	Region Labels	Hemisphere	BA	Vertices	Talairach			t_{max}	$p_{cluster}$
							x	y	z		
60%											
	ASD > Control										
		1	Postcentral gyrus, precentral gyrus	R	1-4	948	8	-33	66	3.47	2.94×10^{-5}
		2	Paracentral lobule, precentral gyrus	L	4	1629	-8	-15	67	3.18	9.25×10^{-5}
	ASD < Control										
		1	Fusiform gyrus, inferior temporal gyrus, lateral occipital cortex, lingual gyrus	L	17-19, 37	3559	-16	-86	-2	-3.79	2.93×10^{-5}
		2	Rostral middle frontal gyrus	R	10	628	34	51	-8	-3.71	2.94×10^{-5}
		3	Inferior parietal cortex, postcentral gyrus, supramarginal gyrus	R	2, 39-40	3658	51	-43	36	-3.29	1.10×10^{-4}
		4	Parahippocampal gyrus	L	35	578	-21	-22	-17	-3.37	3.85×10^{-3}
		5	Caudal middle frontal gyrus, precentral gyrus, rostral middle frontal gyrus	R	4, 6	5220	40	19	42	-3.67	1.14×10^{-2}
30%											
	ASD > Control										
		1	Medial orbital frontal cortex, superior frontal gyrus	R	10-11	2813	13	47	9	2.78	1.53×10^{-4}
	ASD < Control										
		1	Rostral middle frontal gyrus	R	10, 46	1837	38	50	0	-4.10	2.35×10^{-5}
		2	Fusiform gyrus, inferior parietal cortex, inferior temporal gyrus, lateral occipital cortex, lingual gyrus	L	19-20, 37, 39	4943	-17	-86	-2	-3.91	2.35×10^{-5}
		3	Caudal middle frontal gyrus, postcentral gyrus, precentral gyrus, rostral middle frontal gyrus, supramarginal gyrus	R	1-4, 6	7552	46	-21	48	-3.55	2.35×10^{-5}
		4	Parahippocampal gyrus	L	35	1328	-21	-22	-17	-3.59	7.21×10^{-5}
		5	Parahippocampal gyrus	R	35	1423	24	-26	-13	-3.29	1.42×10^{-2}
		6	Pars triangularis	R	45	968	52	27	7	-3.03	4.02×10^{-2}
0%											
	ASD > Control										
		1	Medial orbital frontal cortex, superior frontal gyrus	R	9-11, 32	3208	10	32	-12	3.18	1.90×10^{-5}
	ASD < Control										
		1	Caudal middle frontal gyrus, pars triangularis, rostral middle frontal gyrus	R	6, 10, 45-46	5285	39	45	3	-4.52	1.80×10^{-5}
		2	Inferior temporal gyrus	L	20, 37	979	-54	-47	-15	-3.84	1.80×10^{-5}
		3	Inferior parietal cortex, lateral occipital cortex, middle temporal gyrus	L	19, 21, 37, 39	2616	-41	-71	8	-2.90	1.94×10^{-5}
		4	Fusiform gyrus, lingual gyrus, parahippocampal gyrus	R	19, 35-37	4146	33	-45	-13	-3.96	9.96×10^{-5}

Continuation on the following page

-1mm											
	ASD > Control										
		1	Medial orbital frontal cortex, superior frontal gyrus	R	10-11	1882	8	30	-13	3.37	3.07×10^{-5}
	ASD < Control										
		1	Pars triangularis, rostral middle frontal gyrus, superior frontal gyrus	R	10, 45-46	6548	39	45	4	-4.56	1.39×10^{-5}
		2	Inferior temporal gyrus	L	20, 37	788	-54	-44	-14	-3.44	1.39×10^{-5}
		3	Lateral orbital frontal cortex, pars triangularis	L	45, 47	1026	-45	33	-8	-3.50	1.51×10^{-2}
		4	Middle temporal gyrus, superior temporal gyrus	R	21-22	4559	56	-12	-4	-3.14	3.04×10^{-2}
-2mm											
	ASD > Control										
		1	Medial orbital frontal cortex	R	11	1369	8	32	-13	3.37	3.57×10^{-5}
	ASD < Control										
		1	Pars triangularis, rostral middle frontal gyrus	R	10, 45-46	5012	35	49	-3	-3.92	1.44×10^{-5}
		2	Banks superior temporal sulcus, inferior temporal gyrus, middle temporal gyrus, superior temporal gyrus	R	20-22	3169	57	-27	-22	-3.35	1.49×10^{-5}
		3	Inferior temporal gyrus	L	37	592	-54	-44	-15	-3.25	1.77×10^{-5}
		4	Inferior temporal gyrus	L	20	354	-53	-25	-24	-3.04	2.17×10^{-5}
		5	Lateral orbital frontal cortex, pars triangularis	L	45, 47	1596	-44	32	-6	-3.37	3.46×10^{-5}
		6	Fusiform gyrus, inferior temporal gyrus	R	20, 37	2425	39	-45	-14	-3.91	5.96×10^{-5}
		7	Rostral middle frontal gyrus	L	10	326	-36	49	0	-2.89	1.04×10^{-2}
		8	Postcentral gyrus, precentral gyrus	L	1-4	895	-12	-33	71	-2.75	4.49×10^{-2}

Note: Hemisphere: *L*: Left, *R*: Right; *BA*: approximate Brodmann area(s); Vertices: number of vertices within the cluster; t_{max}: maximum *t*-statistic within the cluster; $p_{cluster}$: cluster-corrected *p*-value.

Supplementary Table S3. Clusters with significantly increased and decreased mean diffusivity (MD) at the gray-white matter boundary (0%) and different sampling depths within the gray matter and superficial white matter

Sampling Depth	Contrast	Cluster	Region Labels	Hemisphere	BA	Vertices	Talairach			t_{max}	$p_{cluster}$
							x	y	z		
60%											
	ASD < Control										
		1	Postcentral gyrus, precentral gyrus	L	1-4	988	-10	-32	68	-3.73	3.84×10^{-5}
		2	Middle temporal gyrus, superior temporal gyrus, transverse temporal cortex	R	21-22, 41	3878	50	-16	-15	-3.41	3.84×10^{-5}
		3	Caudal anterior cingulate cortex, medial orbital frontal cortex, paracentral lobule, posterior cingulate cortex, precuneus cortex, superior frontal gyrus	R	4, 6-7, 9-11, 24, 31-32	8102	10	46	-12	-4.08	3.84×10^{-5}
		4	Middle temporal gyrus, superior temporal gyrus	L	21-22	2612	-55	-17	-15	-3.31	3.85×10^{-5}
		5	Paracentral lobule, posterior cingulate cortex, precuneus cortex, superior frontal gyrus	L	4, 6-7, 24, 31	9160	-11	-3	37	-3.73	3.85×10^{-5}
		6	Superior parietal cortex	R	5	357	12	-43	67	-2.91	4.42×10^{-5}
		7	Postcentral gyrus, precentral gyrus	R	1-4, 6	1744	29	-18	60	-3.33	5.68×10^{-4}
		8	Lateral orbital frontal cortex	L	11, 47	1118	-22	34	-11	-3.16	1.79×10^{-2}
		9	Superior parietal cortex	R	5	628	32	-44	60	-2.73	2.40×10^{-2}
30%											
	ASD > Control										
		1	Pars triangularis	L	45	349	-48	32	-4	2.93	6.33×10^{-4}
	ASD < Control										
		1	Inferior temporal gyrus	R	20	626	49	-14	-31	-3.08	2.95×10^{-5}
		2	Postcentral gyrus	L	3	632	-9	-33	69	-3.72	2.95×10^{-5}
		3	Medial orbital frontal cortex	R	10-11	1906	9	46	-13	-3.44	2.99×10^{-5}
		4	Middle temporal gyrus, superior temporal gyrus	R	21-22	927	51	1	-24	-3.25	9.61×10^{-5}
0%											
	ASD > Control										
		1	Pars triangularis	L	45	970	-48	31	-3	3.06	1.81×10^{-4}
		2	Rostral middle frontal gyrus, superior frontal gyrus	L	10-11	2124	-20	58	6	2.92	1.99×10^{-4}
		3	Superior frontal gyrus	R	10	522	13	62	-3	2.55	9.57×10^{-3}
		4	Fusiform gyrus, inferior temporal gyrus	L	20, 37	627	-49	-32	-20	2.40	2.04×10^{-2}
		5	Fusiform gyrus	R	37	746	49	-32	-17	2.69	2.06×10^{-2}

Continuation on the following page

0%											
	ASD < Control										
		1	Inferior temporal gyrus	R	20	590	45	−11	−32	−2.84	2.25×10^{-5}
		2	Lateral orbital frontal cortex	L	47	456	−18	41	−14	−3.06	2.02×10^{-2}
-1mm											
	ASD > Control										
		1	Medial orbital frontal cortex, rostral middle frontal gyrus, superior frontal gyrus	L	10-11	2706	−12	53	1	2.88	1.14×10^{-4}
		2	Rostral middle frontal gyrus, superior frontal gyrus	R	10	1082	15	62	−2	2.92	6.10×10^{-4}
		3	Fusiform gyrus, inferior temporal gyrus	L	20, 37	864	−49	−32	−20	2.52	5.06×10^{-3}
	ASD < Control										
		1	Lateral orbital frontal cortex	L	11, 47	358	−12	45	−20	−2.98	5.51×10^{-4}
-2mm											
	ASD > Control										
		1	Rostral middle frontal gyrus, superior frontal gyrus	R	10	833	23	56	3	2.51	3.77×10^{-3}

Note: Hemisphere: *L*: Left, *R*: Right; *BA*: approximate Brodmann area(s); Vertices: number of vertices within the cluster; t_{max}: maximum *t*-statistic within the cluster; $p_{cluster}$: cluster-corrected *p*-value.

Supplementary Table S4. Clusters with significantly increased and decreased gray-white matter tissue contrast (GWC)

Contrast	Cluster	Region Labels	Hemisphere	BA	Vertices	Talairach			t_{max}	$p_{cluster}$
						x	y	z		
ASD < Control										
	1	Banks superior temporal sulcus, caudal anterior cingulate cortex, cuneus cortex, entorhinal cortex, fusiform gyrus, inferior parietal cortex, inferior temporal gyrus, insula, isthmus cingulate cortex, lateral occipital cortex, lateral orbital frontal cortex, lingual gyrus, medial orbital frontal cortex, middle temporal gyrus, paracentral lobule, parahippocampal gyrus, pars opercularis, pars orbitalis, pars triangularis, postcentral gyrus, posterior cingulate cortex, precentral gyrus, precuneus cortex, rostral anterior cingulate cortex, rostral middle frontal gyrus, superior frontal gyrus, superior parietal cortex, superior temporal gyrus, supramarginal gyrus, temporal pole	R	1-7, 9-11, 13, 18-21, 23-24, 30-33, 37-41, 44-46	70902	61	-27	-9	-3.70	4.08×10^{-6}
	2	Fusiform gyrus, isthmus cingulate cortex, lingual gyrus, parahippocampal gyrus, precuneus cortex, superior parietal cortex	L	7, 18-19, 30, 37	7051	-12	-66	38	-3.01	4.58×10^{-5}
	3	Postcentral gyrus, superior parietal cortex, supramarginal gyrus	L	1-3, 7, 40	7139	-50	-30	24	-3.43	9.13×10^{-3}
	4	Medial orbital frontal cortex, rostral anterior cingulate cortex, superior frontal gyrus	L	10-11, 24, 32-33	2950	-3	29	-3	-3.69	1.62×10^{-2}

Note: Hemisphere: *L*: Left, *R*: Right; *BA*: approximate Brodmann area(s); Vertices: number of vertices within the cluster; t_{max}: maximum *t*-statistic within the cluster; $p_{cluster}$: cluster-corrected *p*-value.

Supplementary Table S5. Clusters with a significant group-by-sex interaction in fractional anisotropy (FA) at the gray-white matter boundary (0%) and different sampling depths within the gray matter and superficial white matter

Sampling Depth	Contrast	Cluster	Region Labels	Hemisphere	BA	Vertices	Talairach			t_{max}	$p_{cluster}$
							x	y	z		
60%											
	Group*Sex										
		1	Pars triangularis	L	45	462	-51	27	4	2.79	5.19×10^{-3}
		2	Cuneus cortex	L	19	1129	-7	-84	32	-4.56	2.93×10^{-5}
		3	Banks superior temporal sulcus, inferior temporal gyrus, middle temporal gyrus	R	20-22	2923	54	-47	-7	-3.29	3.03×10^{-5}
		4	Middle temporal gyrus	L	20-21	472	-59	-13	-18	-3.24	4.17×10^{-5}
		5	Medial orbital frontal cortex, rostral anterior cingulate cortex	R	11, 24, 32	1693	5	22	-6	-3.11	4.42×10^{-4}
30%											
	Group*Sex										
		1	Cuneus cortex, pericalcarine cortex	L	18-19	2185	-6	-93	15	-4.26	2.35×10^{-5}
		2	Inferior temporal gyrus, lateral occipital cortex	R	18-19, 37	1027	38	-85	-4	-3.11	4.44×10^{-5}
0%											
	Group*Sex										
		1	Cuneus cortex, pericalcarine cortex	L	18-19	2386	-7	-94	14	-3.85	1.80×10^{-5}
-1mm											
	Group*Sex										
		1	Isthmus cingulate cortex, paracentral lobule, posterior cingulate cortex, precentral gyrus, superior frontal gyrus	R	4, 23, 30	5235	4	-13	27	-3.89	2.20×10^{-5}
		2	Pars opercularis, pars triangularis	R	44-45	2919	47	12	15	-2.76	3.12×10^{-3}
		3	Rostral middle frontal gyrus	R	10	480	30	49	-6	-2.95	3.17×10^{-2}
-2mm											
	Group*Sex										
		1	Isthmus cingulate cortex, paracentral lobule, posterior cingulate cortex	R	4, 23-24, 31	2915	4	-13	27	-3.96	1.44×10^{-5}
		2	Caudal middle frontal gyrus, insula, pars opercularis, pars triangularis, postcentral gyrus, precentral gyrus, rostral middle frontal gyrus	L	1-4, 6, 13, 44-46	12385	-44	18	17	-3.32	1.45×10^{-5}
		3	Pars opercularis, pars triangularis, postcentral gyrus, precentral gyrus	R	1-4, 6, 44-45	7168	48	7	3	-3.58	1.56×10^{-5}
		4	Rostral middle frontal gyrus	R	10	490	31	49	-6	-3.04	2.31×10^{-5}
		5	Lateral occipital cortex, lingual gyrus, parahippocampal gyrus, pericalcarine cortex	R	17-19	4172	22	-65	5	-2.95	2.32×10^{-4}
		6	Insula, middle temporal gyrus, superior temporal gyrus	L	13, 21-22	3959	-33	6	-11	-3.07	1.11×10^{-3}
		7	Caudal middle frontal gyrus, precentral gyrus, superior frontal gyrus	R	4, 6, 8	4023	26	18	38	-2.98	2.53×10^{-2}
		8	Inferior parietal cortex, superior parietal cortex	R	7, 39	3562	42	-66	32	-4.30	4.59×10^{-2}

Note: Hemisphere: *L*: Left, *R*: Right; *BA*: approximate Brodmann area(s); Vertices: number of vertices within the cluster; t_{max}: maximum *t*-statistic within the cluster; $p_{cluster}$: cluster-corrected *p*-value.

Supplementary Table S6. Clusters with a significant group-by-sex interaction in mean diffusivity (MD) at the gray-white matter boundary (0%) and different sampling depths within the gray matter and superficial white matter

Sampling Depth	Contrast	Cluster	Region Labels	Hemisphere	BA	Vertices	Talairach			t_{max}	$p_{cluster}$
							x	y	z		
60%											
	Group*Sex										
		1	Rostral middle frontal gyrus	R	10	665	34	48	-4	3.49	3.84×10^{-5}
		2	Medial orbital frontal cortex, superior frontal gyrus	R	11	1844	11	42	-19	3.70	3.84×10^{-5}
		3	Paracentral lobule, posterior cingulate cortex, precuneus cortex	R	4-5, 7, 31	2793	10	-45	64	3.26	1.95×10^{-3}
30%											
	Group*Sex										
		1	Rostral middle frontal gyrus	R	10	747	34	48	-4	3.38	2.95×10^{-5}
		2	Rostral anterior cingulate cortex, superior frontal gyrus	L	10, 24, 32-33	1559	-5	24	15	2.97	3.21×10^{-5}
		3	Precuneus cortex	R	7	660	13	-39	63	2.81	5.99×10^{-4}
		4	Medial orbital frontal cortex, paracentral lobule, posterior cingulate cortex, rostral anterior cingulate cortex, superior frontal gyrus	R	4, 10, 23-24, 32	4601	9	60	-8	2.53	2.97×10^{-2}
0%											
	Group*Sex										
		1	Insula, lateral orbital frontal cortex, pars opercularis, pars orbitalis, pars triangularis, rostral middle frontal gyrus	R	10, 13, 44-45, 47	4533	39	48	-6	3.77	2.24×10^{-5}
		2	Caudal middle frontal gyrus, insula, lateral orbital frontal cortex, pars opercularis, pars triangularis, postcentral gyrus, precentral gyrus, rostral middle frontal gyrus, superior temporal gyrus, supramarginal gyrus, transverse temporal cortex	L	1-4, 6, 13, 40-41, 44-46	21191	-56	0	26	4.11	2.24×10^{-5}
		3	Caudal anterior cingulate cortex, rostral anterior cingulate cortex, superior frontal gyrus	L	10, 24, 32-33	1887	-5	24	15	2.88	2.27×10^{-5}
		4	Posterior cingulate cortex, superior frontal gyrus	L	6, 24, 31-33	3802	-5	12	23	2.88	4.42×10^{-3}

Continuation on the following page

-1mm											
	Group*Sex										
		1	Insula, lateral orbital frontal cortex, pars opercularis, pars orbitalis, pars triangularis, postcentral gyrus, precentral gyrus, rostral middle frontal gyrus, supramarginal gyrus	R	1-4, 10-11, 13, 40, 44-47	6751	33	49	-11	4.12	1.78×10^{-5}
		2	Caudal middle frontal gyrus, insula, lateral orbital frontal cortex, pars opercularis, pars triangularis, postcentral gyrus, precentral gyrus, rostral middle frontal gyrus, superior temporal gyrus, supramarginal gyrus	L	1-4, 6, 9, 13, 40-41, 44-47	24305	-55	6	25	4.70	1.78×10^{-5}
		3	Caudal anterior cingulate cortex, posterior cingulate cortex, superior frontal gyrus	L	6, 23-24, 32-33	4512	-5	24	15	2.91	1.81×10^{-5}
		4	Fusiform gyrus, inferior temporal gyrus	L	20, 37	609	-40	-14	-21	2.75	3.03×10^{-4}
		5	Caudal middle frontal gyrus, pars opercularis, precentral gyrus, rostral middle frontal gyrus, superior frontal gyrus	R	4, 6, 8-9, 44	8778	22	2	57	3.49	1.20×10^{-3}
		6	Inferior parietal cortex, superior parietal cortex, supramarginal gyrus	R	7, 40	8221	39	-43	34	4.41	1.41×10^{-3}
		7	Fusiform gyrus, lingual gyrus, parahippocampal gyrus	L	19, 37	1333	-19	-36	-5	3.07	3.08×10^{-2}
-2mm											
	Group*Sex										
		1	Insula, lateral orbital frontal cortex, pars opercularis, pars orbitalis, pars triangularis, postcentral gyrus, precentral gyrus, rostral middle frontal gyrus, supramarginal gyrus	R	1-4, 10-11, 13, 40, 44-45, 47	7521	33	49	-11	4.25	1.92×10^{-5}
		2	Banks superior temporal sulcus, caudal middle frontal gyrus, insula, lateral orbital frontal cortex, middle temporal gyrus, pars opercularis, pars triangularis, postcentral gyrus, precentral gyrus, rostral middle frontal gyrus, superior temporal gyrus, supramarginal gyrus, transverse temporal cortex	L	1-4, 6, 9, 13, 21-22, 38, 40-41, 44-47	32620	-54	6	25	4.49	1.92×10^{-5}
		3	Caudal anterior cingulate cortex, paracentral lobule, posterior cingulate cortex, rostral anterior cingulate cortex, rostral middle frontal gyrus, superior frontal gyrus	L	4, 6, 8, 10-11, 23-24,32-33	9106	-1	24	-3	3.56	1.92×10^{-5}
		4	Inferior parietal cortex, postcentral gyrus, superior parietal cortex, supramarginal gyrus	R	1-3, 7, 39-40	8296	45	-46	39	3.54	1.01×10^{-4}
		5	Caudal anterior cingulate cortex, medial orbital frontal cortex, rostral anterior cingulate cortex, superior frontal gyrus	R	10, 24, 32-33	1892	13	56	-10	2.88	1.02×10^{-4}
		6	Caudal middle frontal gyrus, pars opercularis, precentral gyrus, rostral middle frontal gyrus, superior frontal gyrus	R	4, 6, 8-9, 44	9941	35	-14	50	3.98	4.59×10^{-4}
		7	Fusiform gyrus, parahippocampal gyrus	L	30, 37	1407	-19	-36	-5	3.17	9.04×10^{-4}

Note: Hemisphere: *L*: Left, *R*: Right; *BA*: approximate Brodmann area(s); Vertices: number of vertices within the cluster; t_{max}: maximum *t*-statistic within the cluster; $p_{cluster}$: cluster-corrected *p*-value.

Supplementary Figures

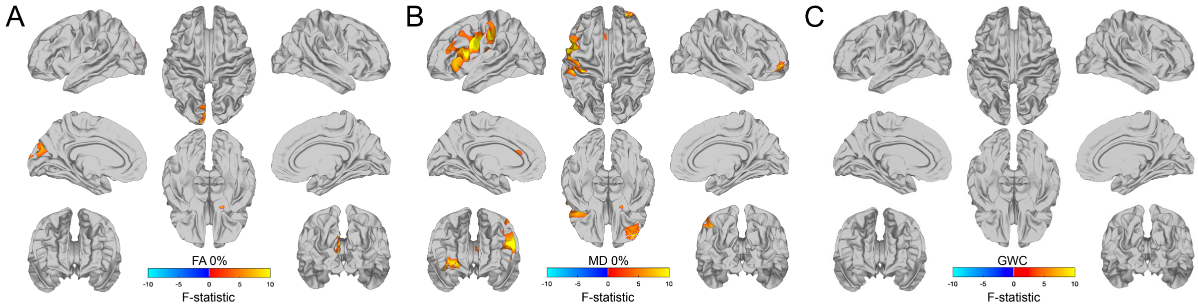

Supplementary Figure S1. ***Nested Model Comparison comparing a general linear model without vs. with a group-by-sex interaction term (N=184).*** Clusters with improvement of model fit by inclusion of a group-by-sex interaction term in the total sample (N=184) for **(A)** fractional anisotropy (FA), **(B)** mean diffusivity (MD), both sampled at the gray-white matter boundary, i.e., at 0% cortical thickness, and for **(C)** gray-white matter tissue contrast (GWC).

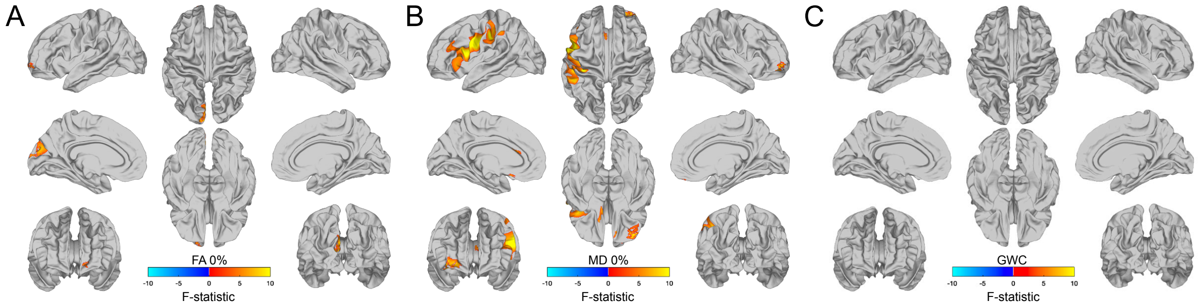

Supplementary Figure S2. ***Nested Model Comparison comparing a general linear model without vs. with a group-by-sex interaction term (N=177).*** Clusters with improvement of model fit by inclusion of a group-by-sex interaction term in all participants, for whom the total score in the Beck Depression Inventory (BDI) was available (N=177), for **(A)** fractional anisotropy (FA), **(B)** mean diffusivity (MD), both sampled at the gray-white matter boundary, i.e., at 0% cortical thickness, and for **(C)** gray-white matter tissue contrast (GWC).

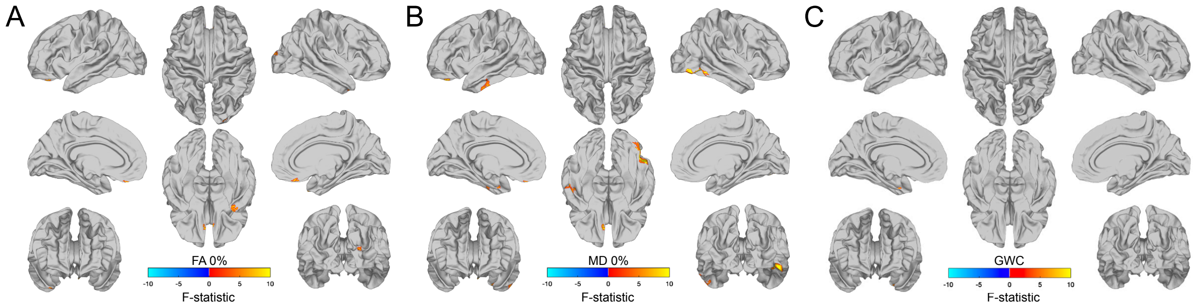

Supplementary Figure S3. ***Nested Model Comparison comparing a general linear model without vs. with the total severity of depressive symptoms (i.e., total score in the Beck Depression Inventory [BDI]) as covariate (N=177).*** Clusters with improvement of model fit by inclusion of the total score in the BDI as covariate in all participants, for whom BDI total score was available (N=177), for **(A)** fractional anisotropy (FA), **(B)** mean diffusivity (MD), both sampled at the gray-white matter boundary, i.e., at 0% cortical thickness, and for **(C)** gray-white matter tissue contrast (GWC).

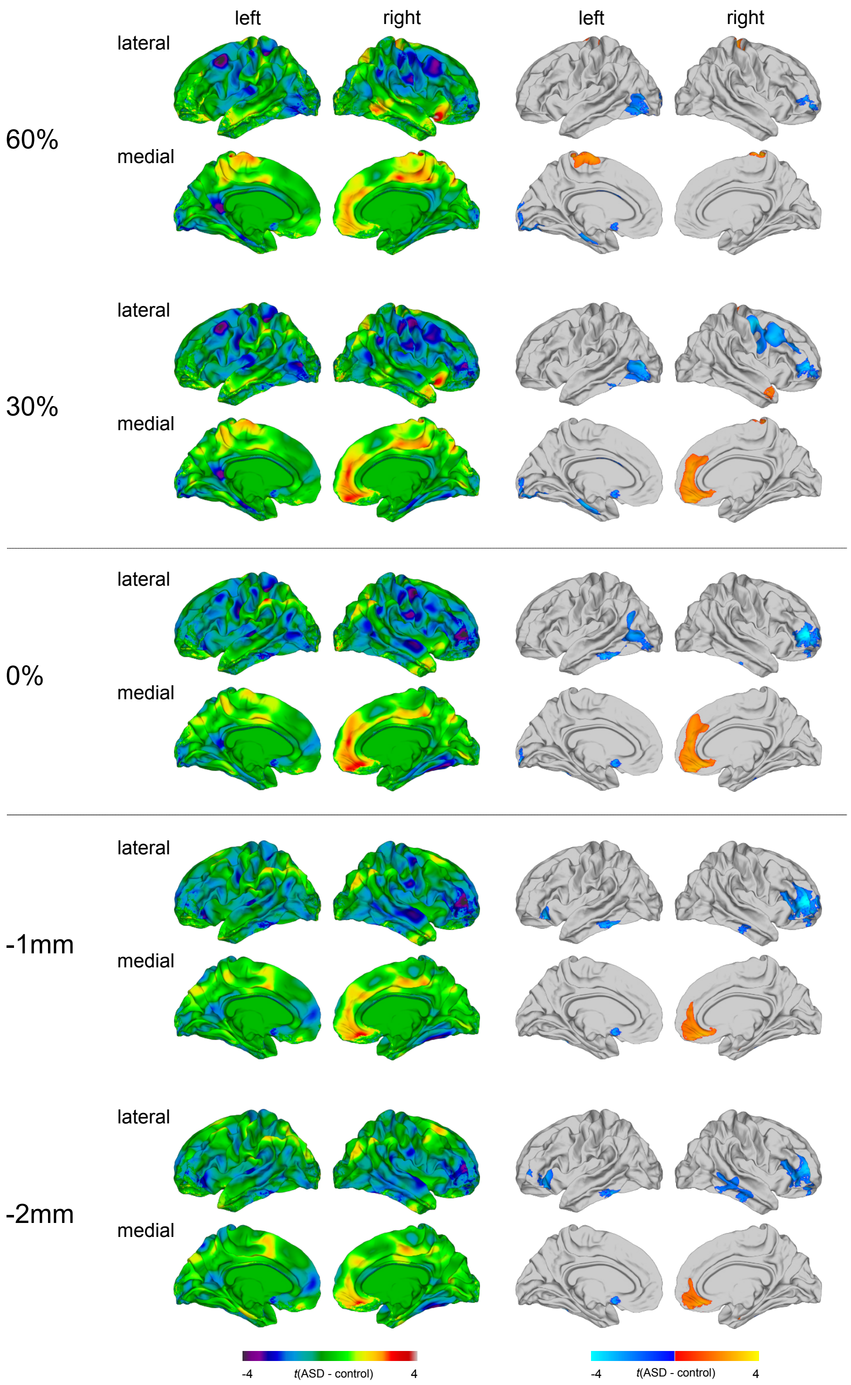

Supplementary Figure S4. ***Main Effect of Group for Fractional Anisotropy (FA) when excluding all participants with a comorbid diagnosis of depression and/or antidepressant medication intake (N=173).*** Regions of increased and decreased FA in individuals with autism spectrum disorder (ASD) compared to typically developing (TD) controls at the gray-white matter (GWM) boundary (0%), at different cortical thickness (CT) projection fractions within the gray matter (i.e., 30% and 60% CT, sampled from the GWM boundary into the thickness of the cortical ribbon), and within the superficial white matter (sampled at absolute distances of -1mm and -2mm below the GWM boundary). Displayed are the unthresholded (left panel) and thresholded (right panel) t-maps, where increased FA estimates in ASD are marked in yellow to red (left panel), respectively, red to yellow (right panel), and decreased FA estimates in ASD are marked in cyan to purple (left panel), respectively, blue to cyan (right panel; random field theory (RFT)-based cluster corrected $p < .05$, two-tailed).

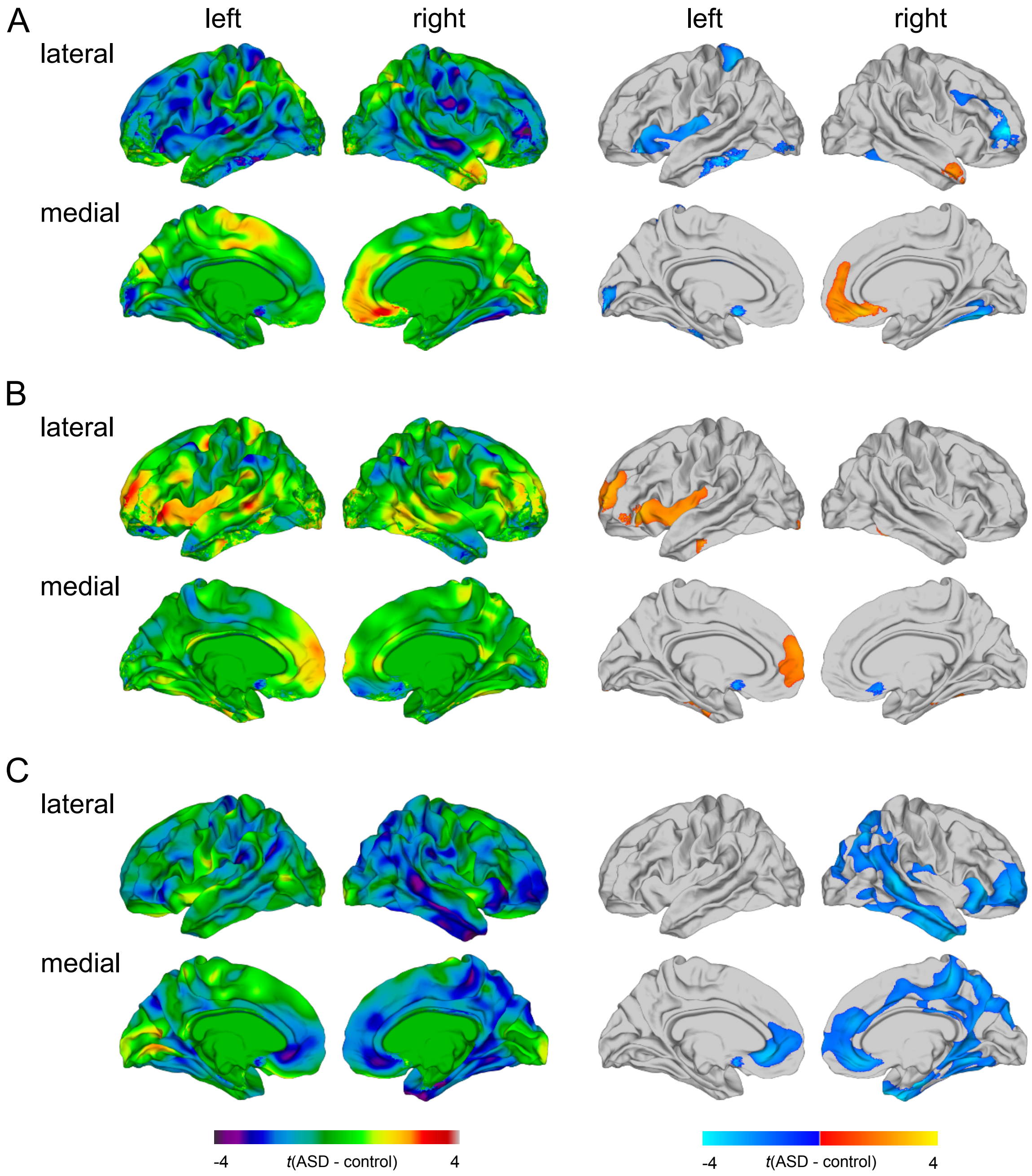

Supplementary Figure S5. ***Main Effect of Group for Fractional Anisotropy (FA), Mean Diffusivity (MD), and gray-white matter tissue contrast (GWC) when covarying for depressive symptoms (N=177).*** Regions of increased and decreased **(A)** FA and **(B)** MD, sampled at the gray-white matter boundary, as well as **(C)** GWC in individuals with autism spectrum disorder (ASD) compared to typically developing (TD) controls, when covarying for depressive symptoms based on the total score in the Beck Depression Inventory (BDI). Displayed are the unthresholded t-maps (left panel) and the random field theory (RFT)-based cluster corrected ($p < .05$, two-tailed) difference maps following multiple comparisons (right panel). Increased parameter estimates in ASD are marked in yellow to red (left panel), respectively, red to yellow (right panel), and decreased parameter estimates in ASD are marked in cyan to purple (left panel), respectively, blue to cyan (right panel).

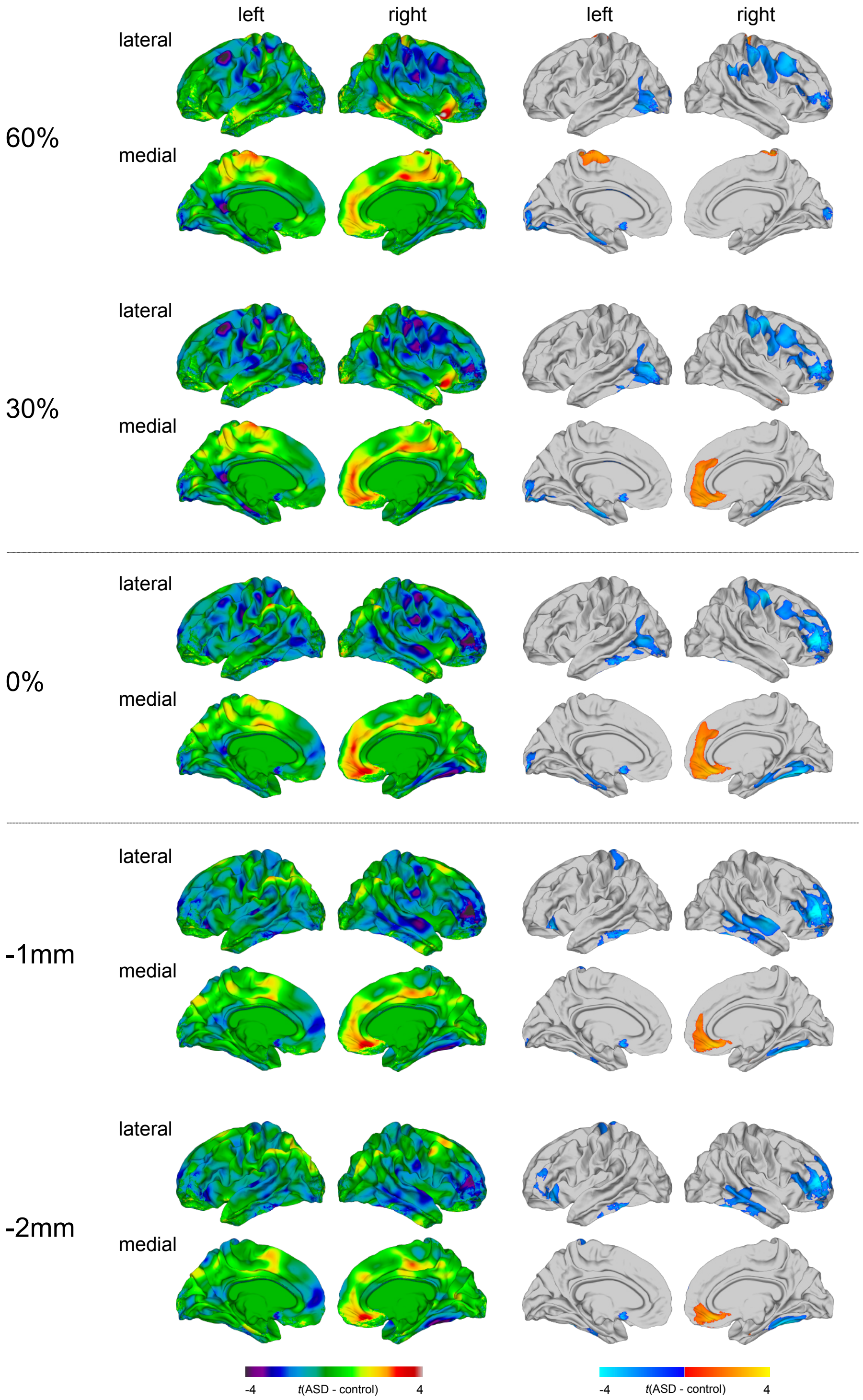

Supplementary Figure S6. *Main Effect of Group for Fractional Anisotropy (FA) when covarying for total gray and white matter volumes.* Regions of increased and decreased FA in individuals with autism spectrum disorder (ASD) compared to typically developing (TD) controls at the gray-white matter (GWM) boundary (0%), at different cortical thickness (CT) projection fractions within the gray matter (i.e., 30% and 60% CT, sampled from the GWM boundary into the thickness of the cortical ribbon), and within the superficial white matter (sampled at absolute distances of −1mm and −2mm below the GWM boundary), when covarying for total gray and white matter volumes. Displayed are the unthresholded (left panel) and thresholded (right panel) *t*-maps, where increased FA estimates in ASD are marked in yellow to red (left panel), respectively, red to yellow (right panel), and decreased FA estimates in ASD are marked in cyan to purple (left panel), respectively, blue to cyan (right panel; random field theory (RFT)-based cluster corrected $p < .05$, two-tailed).

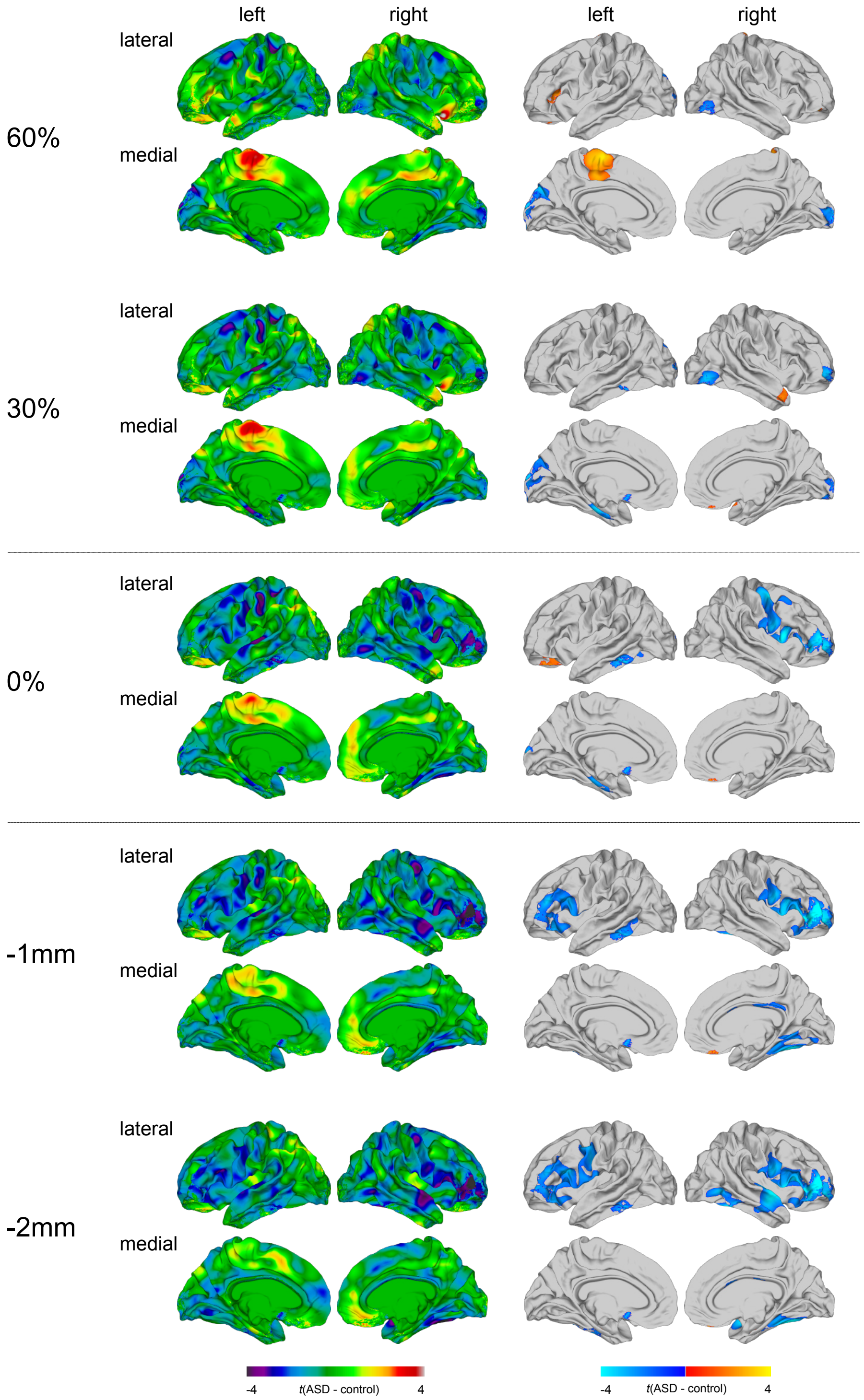

Supplementary Figure S7. ***Main Effect of Group for Fractional Anisotropy (FA) in males only (N=104).*** Regions of increased and decreased FA in male individuals with autism spectrum disorder (ASD) compared to typically developing (TD) male controls at the gray-white matter (GWM) boundary (0%), at different cortical thickness (CT) projection fractions within the gray matter (i.e., 30% and 60% CT, sampled from the GWM boundary into the thickness of the cortical ribbon), and within the superficial white matter (sampled at absolute distances of −1mm and −2mm below the GWM boundary). Displayed are the unthresholded (left panel) and thresholded (right panel) t-maps, where increased FA estimates in ASD are marked in yellow to red (left panel), respectively, red to yellow (right panel), and decreased FA estimates in ASD are marked in cyan to purple (left panel), respectively, blue to cyan (right panel; random field theory (RFT)-based cluster corrected $p < .05$, two-tailed).

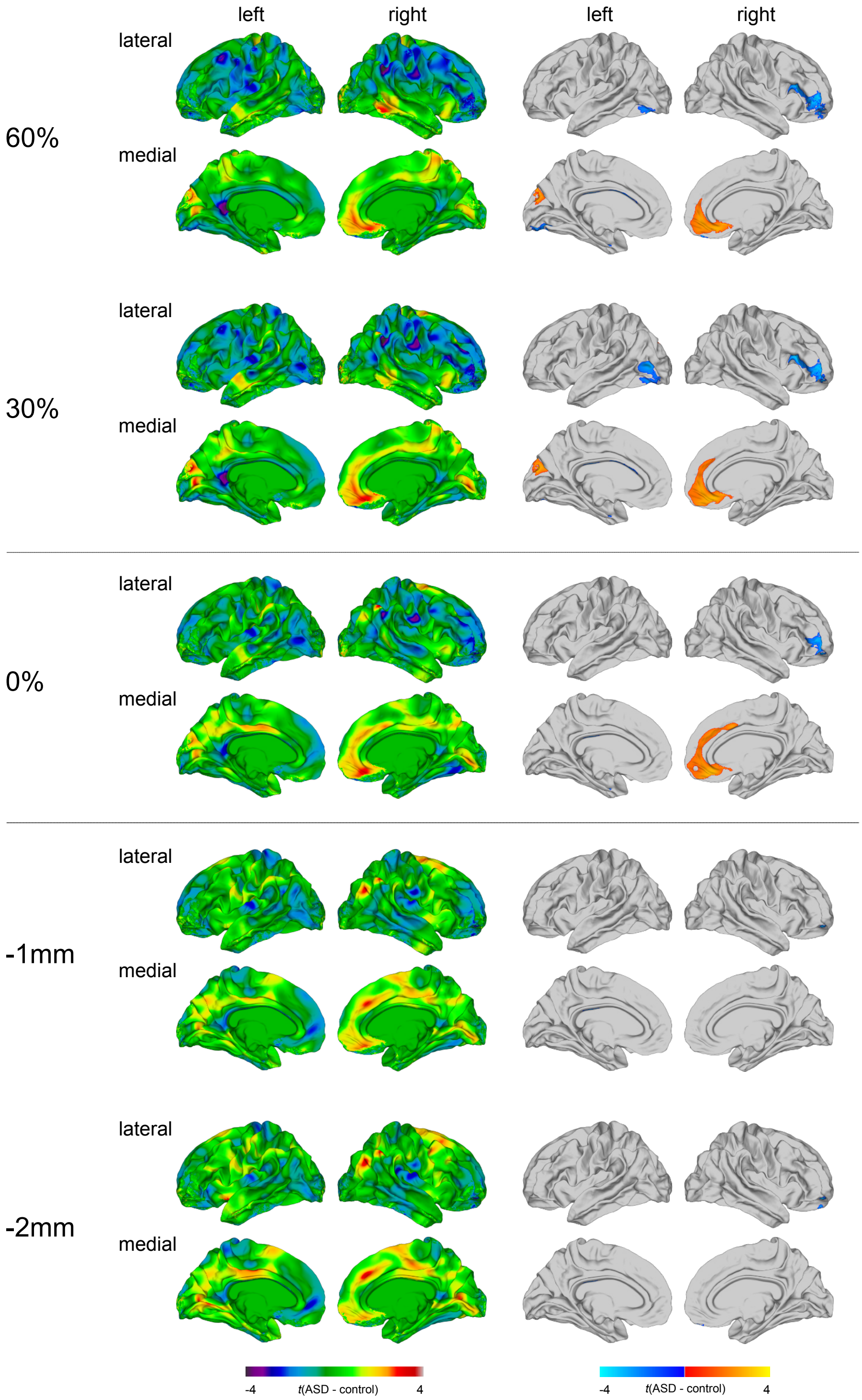

Supplementary Figure S8. ***Main Effect of Group for Fractional Anisotropy (FA) in females only (N=80).*** Regions of increased and decreased FA in female individuals with autism spectrum disorder (ASD) compared to typically developing (TD) female controls at the gray-white matter (GWM) boundary (0%), at different cortical thickness (CT) projection fractions within the gray matter (i.e., 30% and 60% CT, sampled from the GWM boundary into the thickness of the cortical ribbon), and within the superficial white matter (sampled at absolute distances of −1mm and −2mm below the GWM boundary). Displayed are the unthresholded (left panel) and thresholded (right panel) t-maps, where increased FA estimates in ASD are marked in yellow to red (left panel), respectively, red to yellow (right panel), and decreased FA estimates in ASD are marked in cyan to purple (left panel), respectively, blue to cyan (right panel; random field theory (RFT)-based cluster corrected $p < .05$, two-tailed).

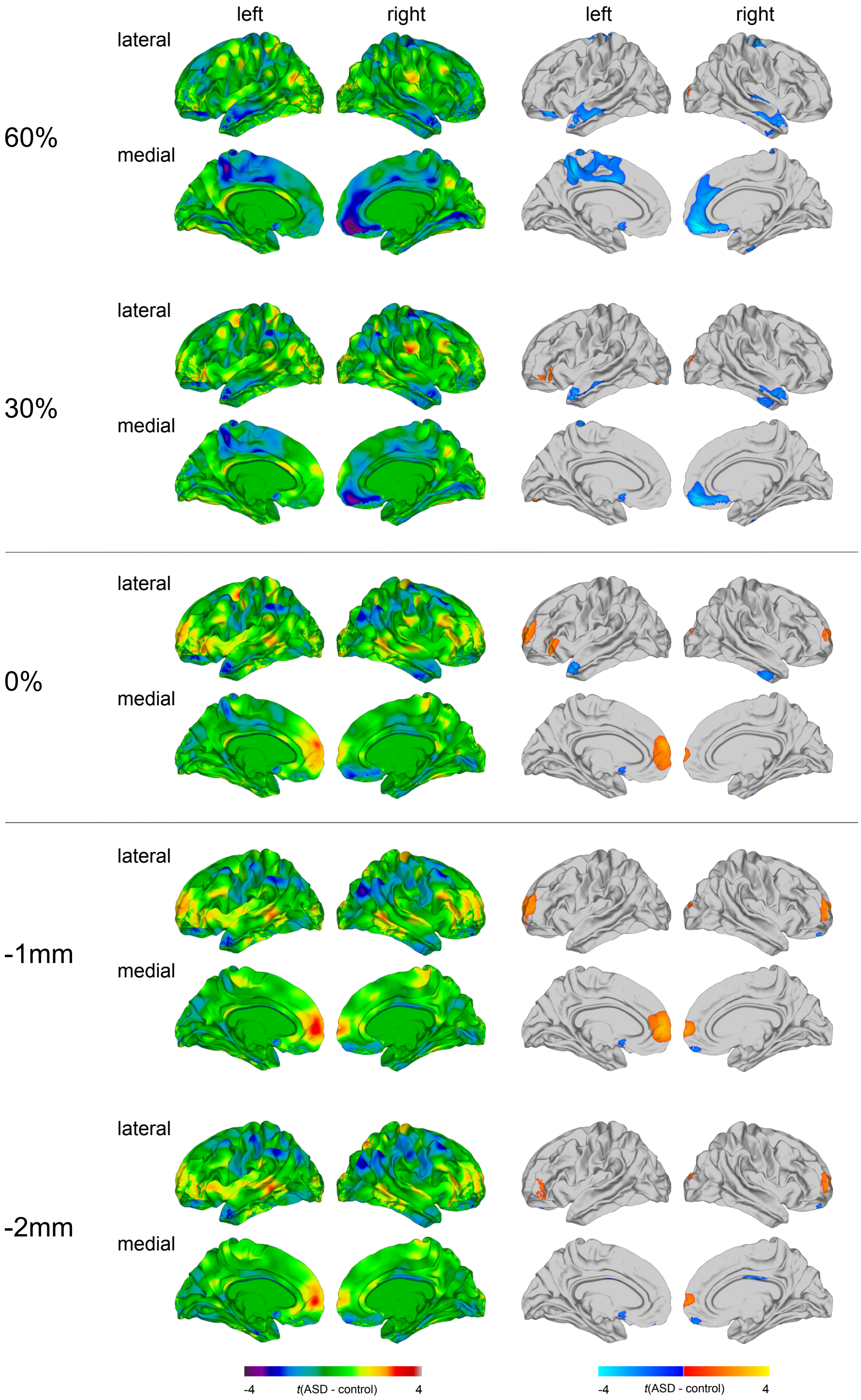

Supplementary Figure S9. *Main Effect of Group for Mean Diffusivity (MD) when excluding all participants with a comorbid diagnosis of depression and/or antidepressant medication intake (N=173).* Regions of increased and decreased MD in individuals with autism spectrum disorder (ASD) compared to typically developing (TD) controls at the gray-white matter (GWM) boundary (0%), at different cortical thickness (CT) projection fractions within the gray matter (i.e., 30% and 60% CT, sampled from the GWM boundary into the thickness of the cortical ribbon), and within the superficial white matter (sampled at absolute distances of -1mm and -2mm below the GWM boundary). Displayed are the unthresholded (left panel) and thresholded (right panel) *t*-maps, where increased MD estimates in ASD are marked in yellow to red (left panel), respectively, red to yellow (right panel), and decreased MD estimates in ASD are marked in cyan to purple (left panel), respectively, blue to cyan (right panel; random field theory (RFT)-based cluster corrected $p < .05$, two-tailed).

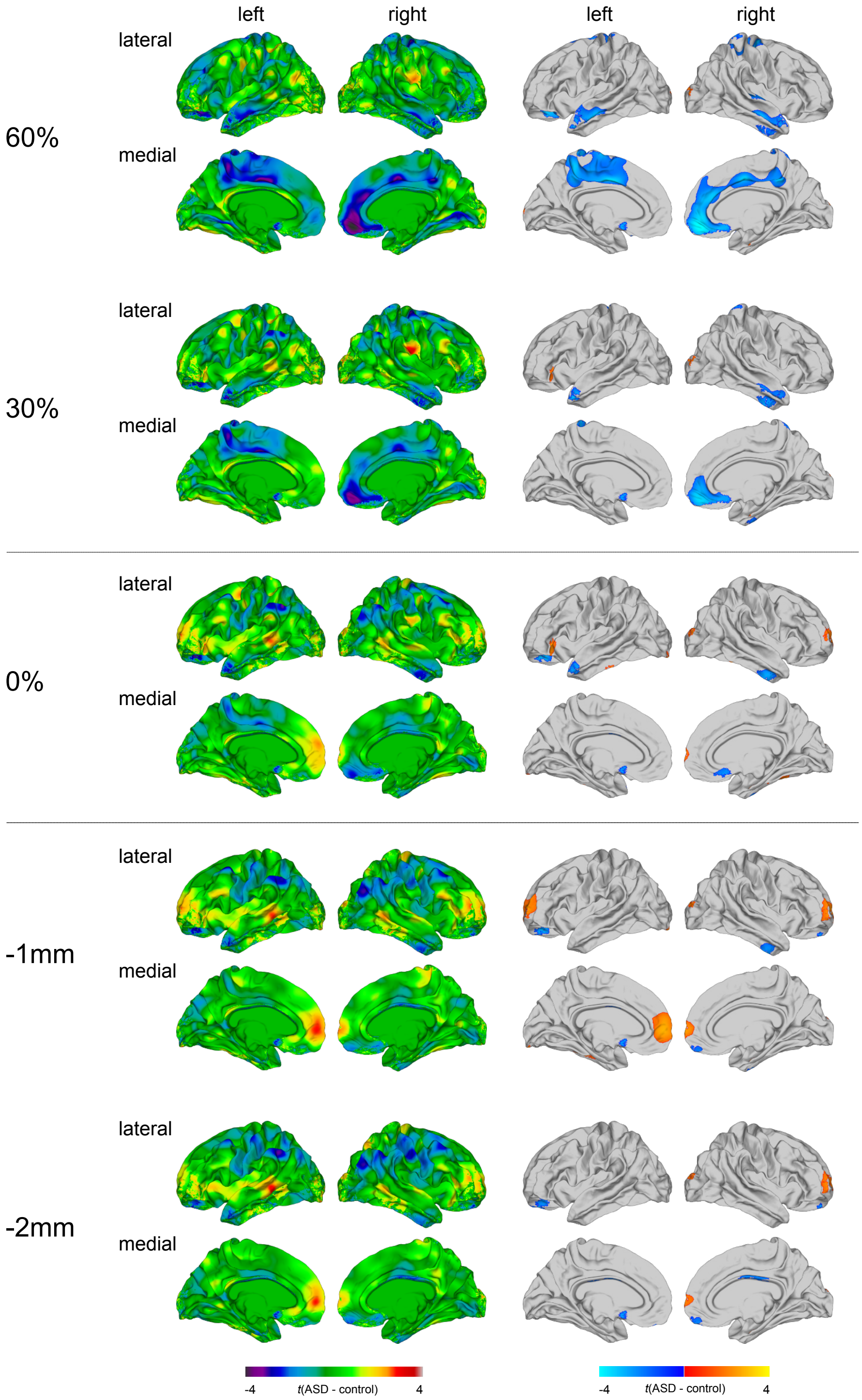

Supplementary Figure S10. ***Main Effect of Group for Mean Diffusivity (MD) when covarying for total gray and white matter volumes.*** Regions of increased and decreased MD in individuals with autism spectrum disorder (ASD) compared to typically developing (TD) controls at the gray-white matter (GWM) boundary (0%), at different cortical thickness (CT) projection fractions within the gray matter (i.e., 30% and 60% CT, sampled from the GWM boundary into the thickness of the cortical ribbon), and within the superficial white matter (sampled at absolute distances of -1mm and -2mm below the GWM boundary), when covarying for total gray and white matter volumes. Displayed are the unthresholded (left panel) and thresholded (right panel) t-maps, where increased MD estimates in ASD are marked in yellow to red (left panel), respectively, red to yellow (right panel), and decreased MD estimates in ASD are marked in cyan to purple (left panel), respectively, blue to cyan (right panel; random field theory (RFT)-based cluster corrected $p < .05$, two-tailed).

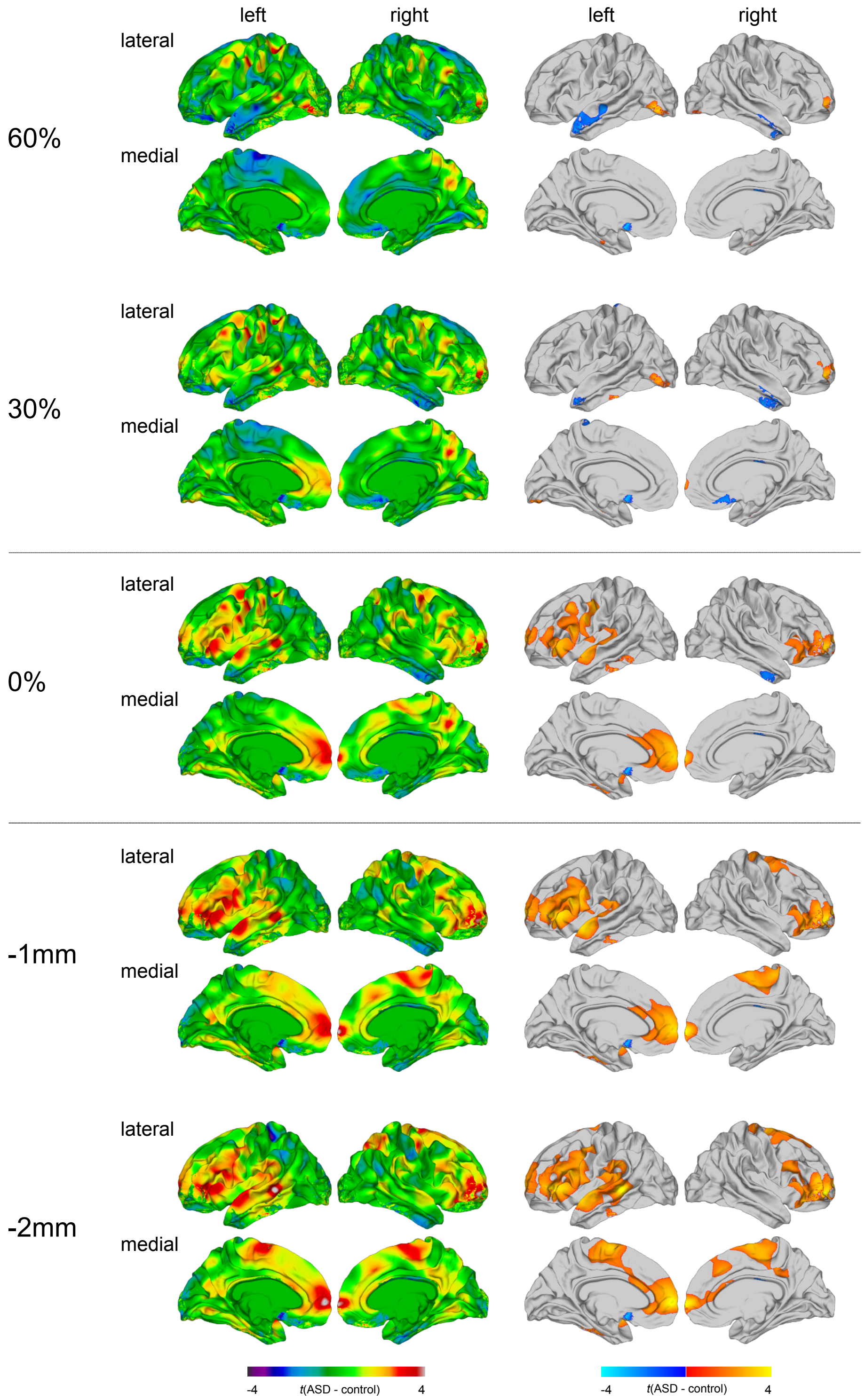

Supplementary Figure S11. *Main Effect of Group for Mean Diffusivity (MD) in males only (N=104).* Regions of increased and decreased MD in male individuals with autism spectrum disorder (ASD) compared to typically developing (TD) male controls at the gray-white matter (GWM) boundary (0%), at different cortical thickness (CT) projection fractions within the gray matter (i.e., 30% and 60% CT, sampled from the GWM boundary into the thickness of the cortical ribbon), and within the superficial white matter (sampled at absolute distances of −1mm and −2mm below the GWM boundary). Displayed are the unthresholded (left panel) and thresholded (right panel) t-maps, where increased MD estimates in ASD are marked in yellow to red (left panel), respectively, red to yellow (right panel), and decreased MD estimates in ASD are marked in cyan to purple (left panel), respectively, blue to cyan (right panel; random field theory (RFT)-based cluster corrected $p < .05$, two-tailed).

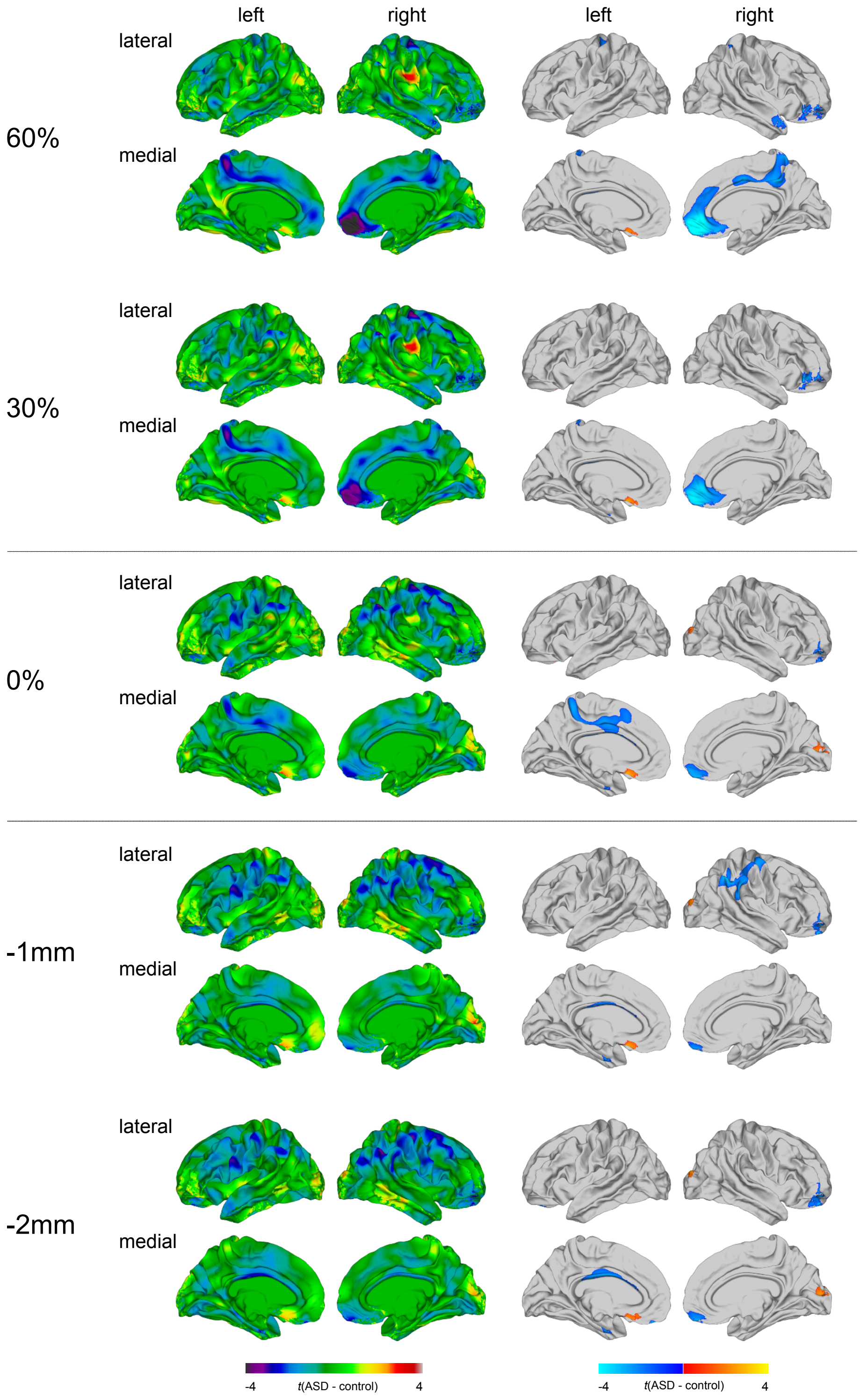

Supplementary Figure S12. *Main Effect of Group for Mean Diffusivity (MD) in females only (N=80).* Regions of increased and decreased MD in female individuals with autism spectrum disorder (ASD) compared to typically developing (TD) female controls at the gray-white matter (GWM) boundary (0%), at different cortical thickness (CT) projection fractions within the gray matter (i.e., 30% and 60% CT, sampled from the GWM boundary into the thickness of the cortical ribbon), and within the superficial white matter (sampled at absolute distances of −1mm and −2mm below the GWM boundary). Displayed are the unthresholded (left panel) and thresholded (right panel) *t*-maps, where increased MD estimates in ASD are marked in yellow to red (left panel), respectively, red to yellow (right panel), and decreased MD estimates in ASD are marked in cyan to purple (left panel), respectively, blue to cyan (right panel; random field theory (RFT)-based cluster corrected $p < .05$, two-tailed).

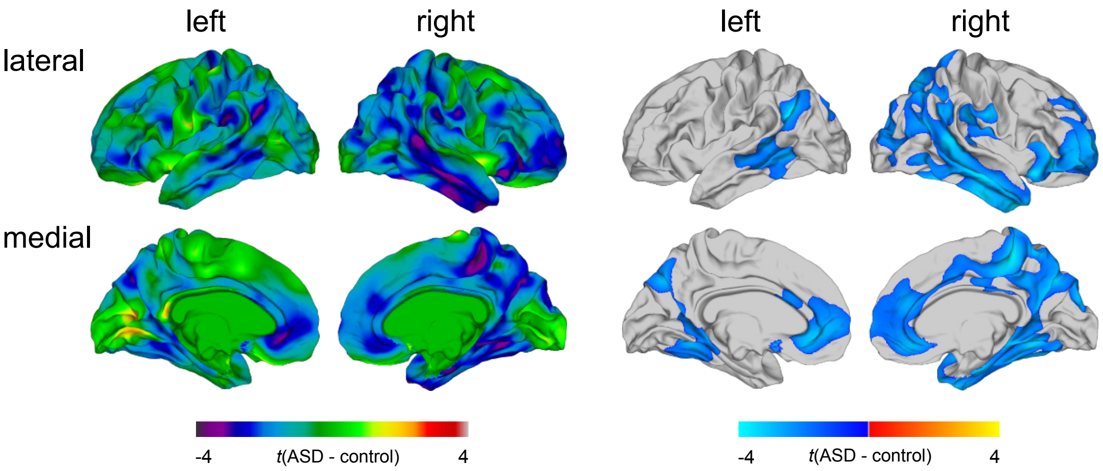

Supplementary Figure S13. ***Main Effect of Group for gray-white matter tissue contrast (GWC) when excluding all participants with a comorbid diagnosis of depression and/or antidepressant medication intake (N=173).*** Regions of significantly reduced GWC in individuals with autism spectrum disorder (ASD) compared to typically developing (TD) controls sampled as ratio between the gray matter signal intensity sampled 30% into the thickness of the cortical ribbon from the gray-white matter (GWM) boundary and white matter signal intensity sampled -1mm below the GWM boundary. Displayed are the unthresholded (left panel) and thresholded (right panel; random field theory (RFT)-based cluster corrected $p < .05$, two-tailed) t-maps.

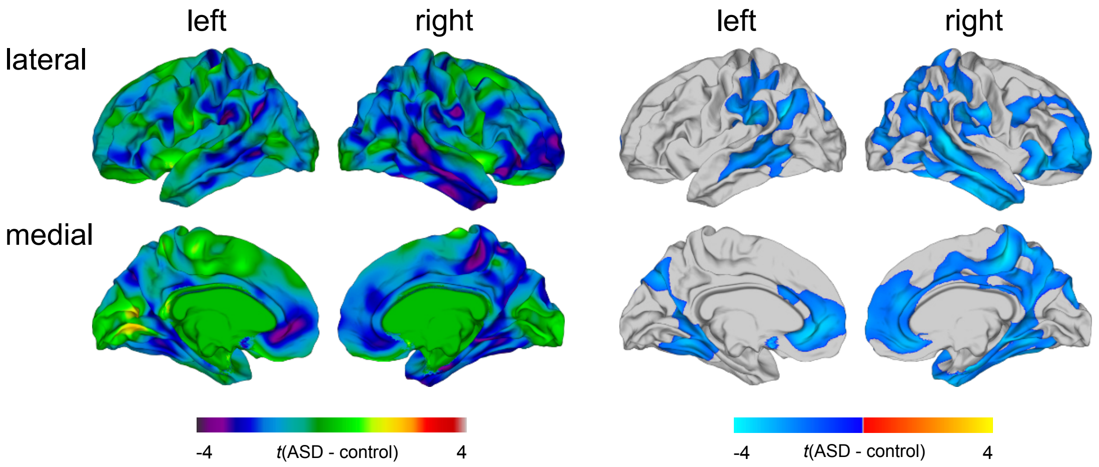

Supplementary Figure S14. ***Main Effect of Group for gray-white matter tissue contrast (GWC) when covarying for total gray and white matter volumes.*** Regions of significantly reduced GWC in individuals with autism spectrum disorder (ASD) compared to typically developing (TD) controls sampled as ratio between the gray matter signal intensity sampled 30% into the thickness of the cortical ribbon from the gray-white matter (GWM) boundary and white matter signal intensity sampled -1mm below the GWM boundary, when covarying for total gray and white matter volumes. Displayed are the unthresholded (left panel) and thresholded (right panel; random field theory (RFT)-based cluster corrected $p < .05$, two-tailed) t-maps.

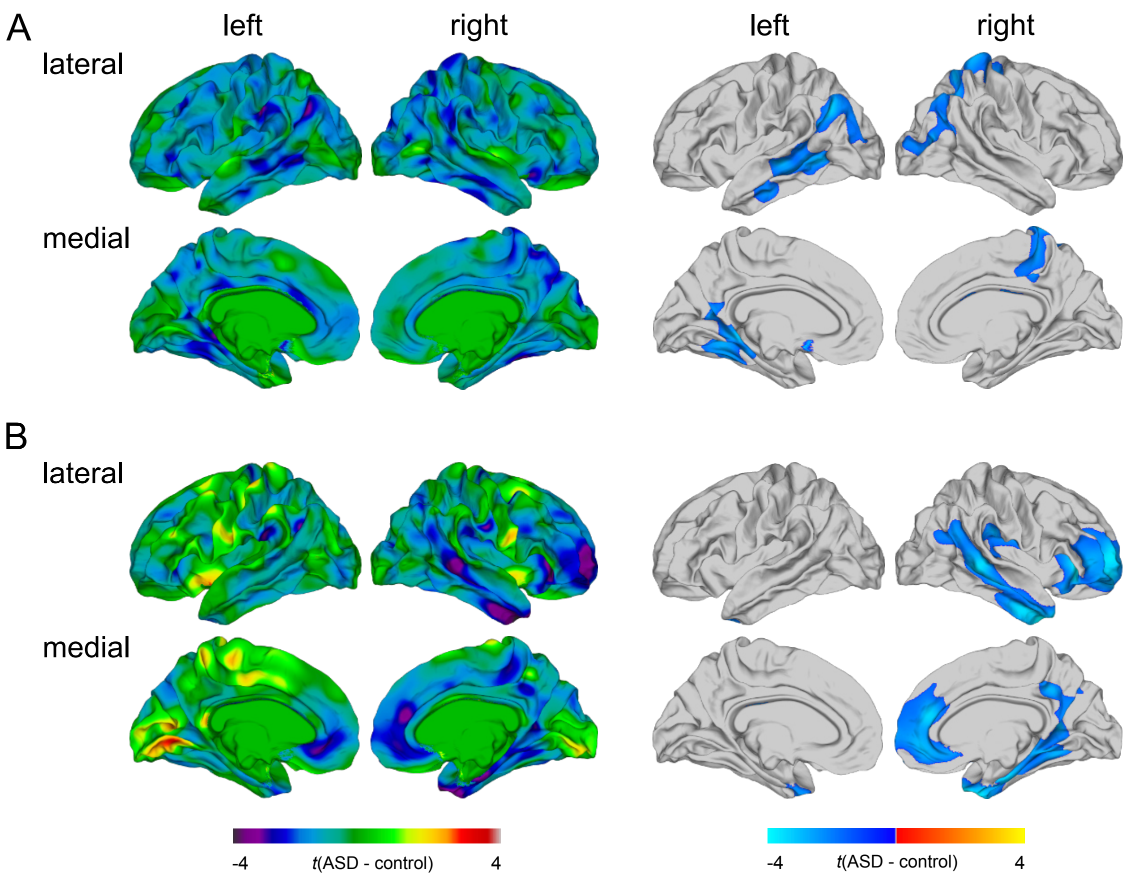

Supplementary Figure S15. ***Main Effect of Group for gray-white matter tissue contrast (GWC) stratified by sex.*** (A) Regions of significantly reduced GWC in male individuals with autism spectrum disorder (ASD) compared to typically developing (TD) male controls sampled as ratio between the gray matter signal intensity sampled 30% into the thickness of the cortical ribbon from the gray-white matter (GWM) boundary and white matter signal intensity sampled -1mm below the GWM boundary. Displayed are the unthresholded (left panel) and thresholded (right panel; random field theory (RFT)-based cluster corrected $p < .05$, two-tailed) t-maps. **(B)** Regions of significantly reduced GWC in female individuals with ASD compared to female TD controls sampled as ratio between the gray matter signal intensity sampled 30% into the thickness of the cortical ribbon from the GWM boundary and white matter signal intensity sampled -1mm below the GWM boundary. Displayed are the unthresholded (left panel) and thresholded (right panel; RFT-based cluster corrected $p < .05$, two-tailed) t-maps.

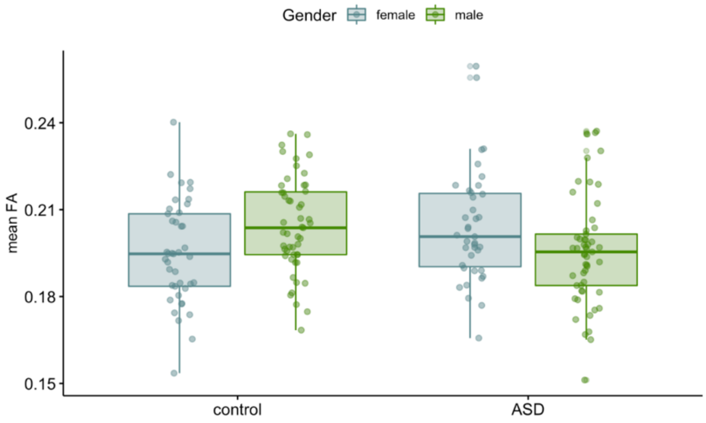

Supplementary Figure S16. ***Boxplots for the significant group-by-sex interaction cluster for fractional anisotropy (FA) sampled at the gray-white matter boundary.*** Boxplots displaying the significant group-by-sex interaction effect for FA in the left cuneus and pericalcarine cortex (Brodmann Area (BA) 17-19). The solid bars indicate the median values of each group, with the lower and upper hinges corresponding to the first (the 25th percentile) and third (the 75th percentile) quartiles. 'ASD' represents individuals with autism spectrum disorder and 'Control' represents typically developing controls.

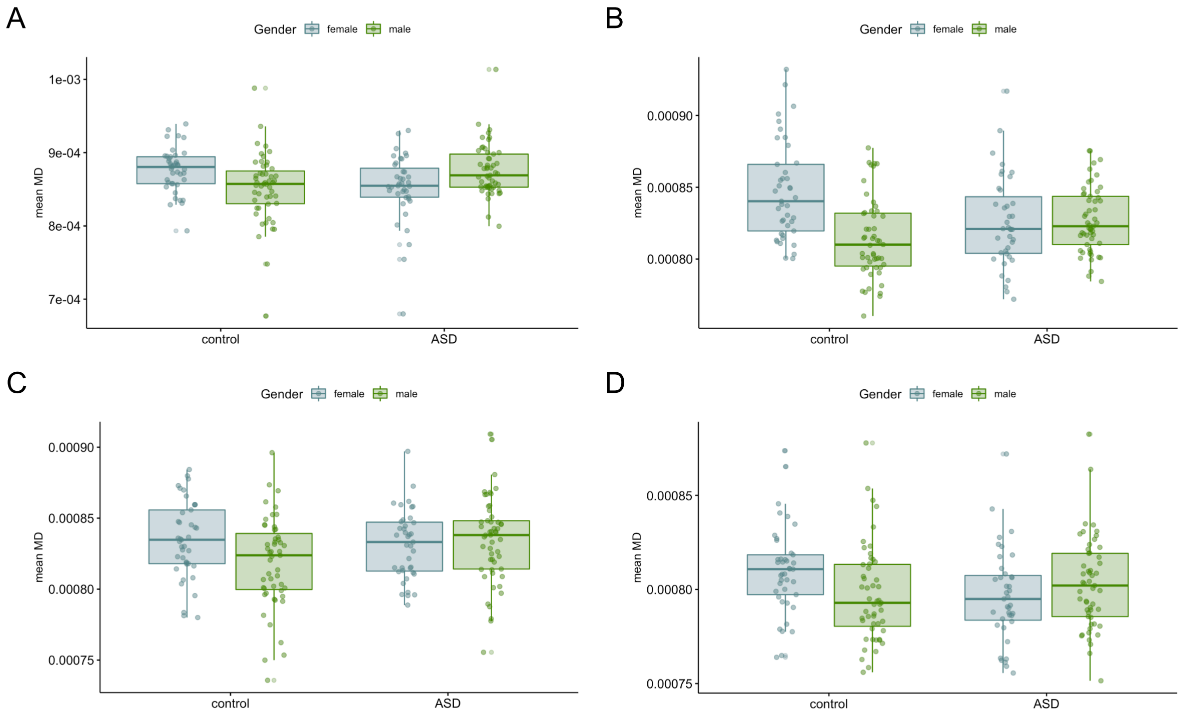

Supplementary Figure S17. ***Boxplots for the significant group-by-sex interaction clusters for mean diffusivity (MD) sampled at the gray-white matter boundary.*** Boxplots displaying the significant group-by-sex interaction effects for MD in **(A)** the right orbitofrontal and ventrolateral prefrontal cortex (Brodmann Area (BA) 10/13/44-45/47); **(B)** left fronto-central and fronto-parietal regions (BA 1-4/6/13/40-41/44-46); **(C)** the left anterior cingulate cortex and superior frontal gyrus (BA 10/24/32-33); and **(D)** the left posterior cingulate cortex and superior frontal gyrus (BA 6/24/31-33). The solid bars indicate the median values of each group, with the lower and upper hinges corresponding to the first (the 25th percentile) and third (the 75th percentile) quartiles. 'ASD' represents individuals with autism spectrum disorder and 'Control' represents typically developing controls.

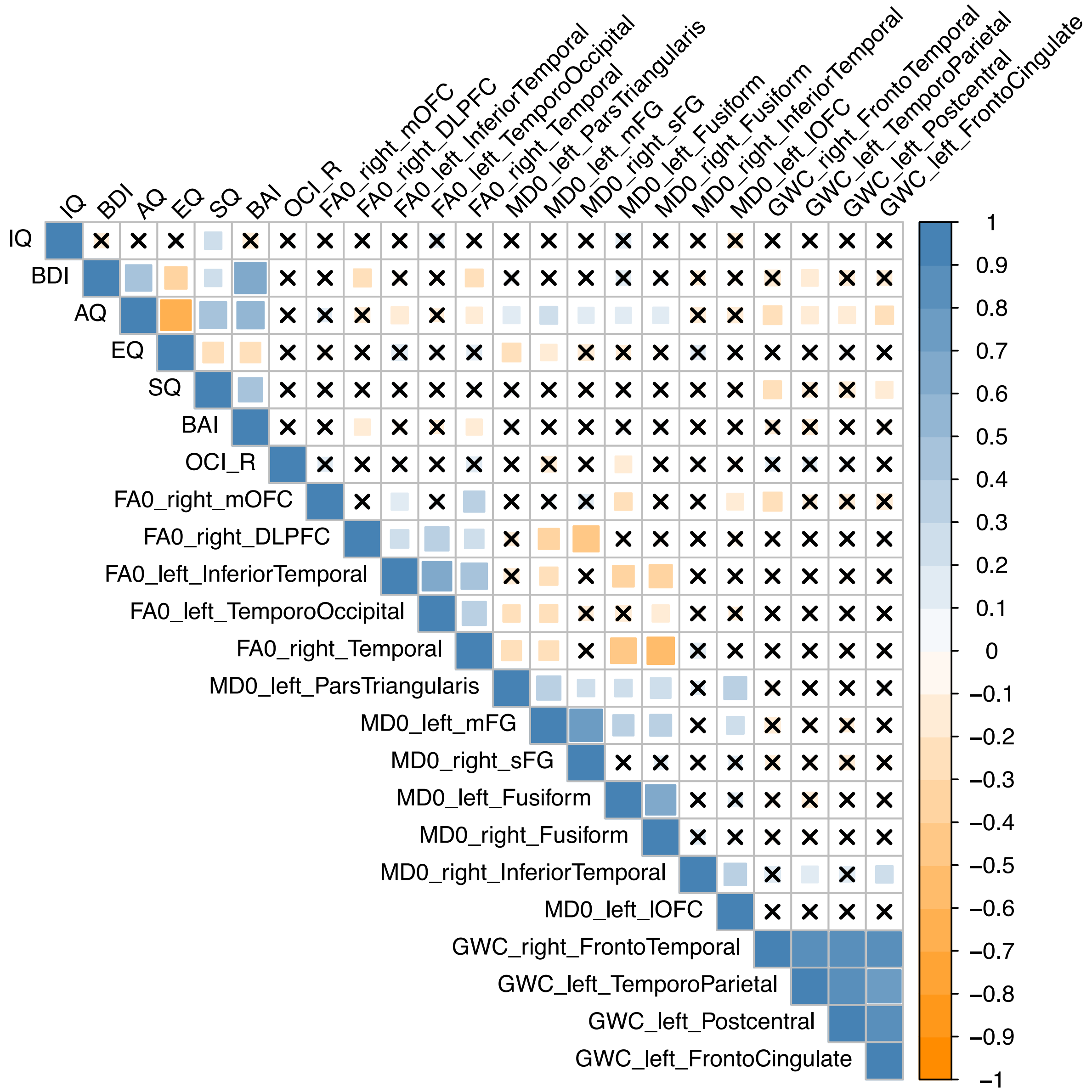

Supplementary Figure S18. ***Association between atypical diffusion and reduced gray-white matter tissue contrast in autism spectrum disorder with clinical symptom severity.*** Correlation Plot depicting Pearson correlation coefficients between participants' full-scale intelligence quotient (IQ), total scores in the distinct questionnaires (i.e., Beck Depression Inventory (BDI), Autism Spectrum Quotient (AQ), Empathy Quotient (EQ), Systemizing Quotient (SQ), Beck Anxiety Inventory (BAI), and Obsessive-Compulsive Inventory-Revised (OCI-R)), and mean cluster values of the clusters obtained in the main effect of group analyses (autism spectrum disorder (ASD) vs. typically developing controls (TDC)) for fractional anisotropy (FA) and mean diffusivity (MD), sampled at the gray-white matter boundary (i.e., FA0 and MD0), as well as gray-white matter tissue contrast (GWC). Multiple testing was addressed by false discovery rate (FDR)-adjustment of *p*-values (one-tailed, $p < .05$). Correlations were based on N=169 participants (n=83 ASD and n=86 TDC), for whom the complete set of questionnaire information was available. Abbreviations: mOFC= medial orbitofrontal cortex; DLPFC= dorsolateral prefrontal cortex; mFG= middle frontal gyrus; sFG= superior frontal gyrus; lOFC= lateral orbitofrontal cortex.

References

Abramowitz, J. S., & Deacon, B. J. (2006). Psychometric properties and construct validity of the Obsessive–Compulsive Inventory—Revised: Replication and extension with a clinical sample. *Journal of Anxiety Disorders*, *20*(8), 1016–1035. https://doi.org/10.1016/j.janxdis.2006.03.001

Baron-Cohen, S., Richler, J., Bisarya, D., Gurunathan, N., & Wheelwright, S. (2003). The systemizing quotient: an investigation of adults with Asperger syndrome or high–functioning autism, and normal sex differences. *Philosophical Transactions of the Royal Society of London B: Biological Sciences, 358*(1430), 361-374. doi:10.1098/rstb.2002.1206

Baron-Cohen, S., & Wheelwright, S. (2004). The empathy quotient: an investigation of adults with Asperger syndrome or high functioning autism, and normal sex differences. *Journal of Autism and Developmental Disorders*, *34*(2), 163-175. doi: 10.1023/b:jadd.0000022607.19833.00

Baron-Cohen, S., Wheelwright, S., Skinner, R., Martin, J., & Clubley, E. (2001). The autism-spectrum quotient (AQ): evidence from Asperger syndrome/high-functioning autism, males and females, scientists and mathematicians. *Journal of Autism and Developmental Disorders, 31*(1), 5-17. doi: 10.1023/a:1005653411471

Beck, A.T., & Steer, R.A. (1987). *Manual for the Beck Depression Inventory*. San Antonio, TX: The Psychological Corporation.

Beck, A. T., & Steer, R. A. (1993). *Beck Anxiety Inventory manual*. San Antonio, TX: Psychological Corporation.

Huppert, J. D., Walther, M. R., Hajcak, G., Yadin, E., Foa, E. B., Simpson, H. B., & Liebowitz, M. R. (2007). The OCI-R: Validation of the subscales in a clinical sample. *Journal of Anxiety Disorders*, *21*(3), 394-406. https://doi.org/10.1016/j.janxdis.2006.05.006
